# Evidence Accumulation and Choice Maintenance Are Dissociated in Human Perceptual Decision Making

**DOI:** 10.1371/journal.pone.0140361

**Published:** 2015-10-28

**Authors:** Mads Lund Pedersen, Tor Endestad, Guido Biele

**Affiliations:** 1 Department of Psychology, University of Oslo, 0317, Oslo, Norway; 2 Intervention Centre, Oslo University Hospital, Rikshospitalet, 0372, Oslo, Norway; 3 Norwegian Institute of Public Health, 0473, Oslo, Norway; University of Groningen, NETHERLANDS

## Abstract

Perceptual decision making in monkeys relies on decision neurons, which accumulate evidence and maintain choices until a response is given. In humans, several brain regions have been proposed to accumulate evidence, but it is unknown if these regions also maintain choices. To test if accumulator regions in humans also maintain decisions we compared delayed and self-paced responses during a face/house discrimination decision making task. Computational modeling and fMRI results revealed dissociated processes of evidence accumulation and decision maintenance, with potential accumulator activations found in the dorsomedial prefrontal cortex, right inferior frontal gyrus and bilateral insula. Potential maintenance activation spanned the frontal pole, temporal gyri, precuneus and the lateral occipital and frontal orbital cortices. Results of a quantitative reverse inference meta-analysis performed to differentiate the functions associated with the identified regions did not narrow down potential accumulation regions, but suggested that response-maintenance might rely on a verbalization of the response.

## Introduction

Perceptual decisions like for example discriminating between toxic and edible mushrooms are well described by sequential sampling models (SSM) of decision making [1,2]. According to such models, choices are made by accumulating perceptual evidence until a decision boundary is reached. Neurophysiological recordings in monkeys have identified the lateral intraparietal area (LIP) within the intraparietal sulcus (IPS) as an accumulator area during perceptual decision making [3,4], along with the frontal eye fields [5], superior colliculus [6] and dorsolateral prefrontal cortex (dlPFC) [7]. The firing rate of these accumulator neurons gradually ramps up until reaching a decision boundary, upon which a motor response is executed. The speed with which a boundary is reached depends on the evidence quality, such that the boundary is crossed earlier for easier decisions. When responses are triggered by a delayed cue, firing in accumulator neurons is sustained at boundary level until the response is executed [3,7,8]. Decision neurons in monkeys thus have multiple functions: accumulating evidence, maintaining choices, and planning motor execution.

Inspired by and in parallel with neurophysiological studies, functional Magnetic Resonance Imaging (fMRI) studies of decision making have identified potential accumulator regions in humans, only some of which are consistent with monkey literature. In addition to the IPS [9–11], the proposed areas include left dlPFC [12,13], right insula [14], left inferior frontal cortex [15] and dorsomedial prefrontal cortex (dmPFC) [11]. While identifying the location of human accumulator regions has received much attention, it remains unexplored whether a human accumulation region also maintains choices, as is found in monkeys.

**Fig 1:**
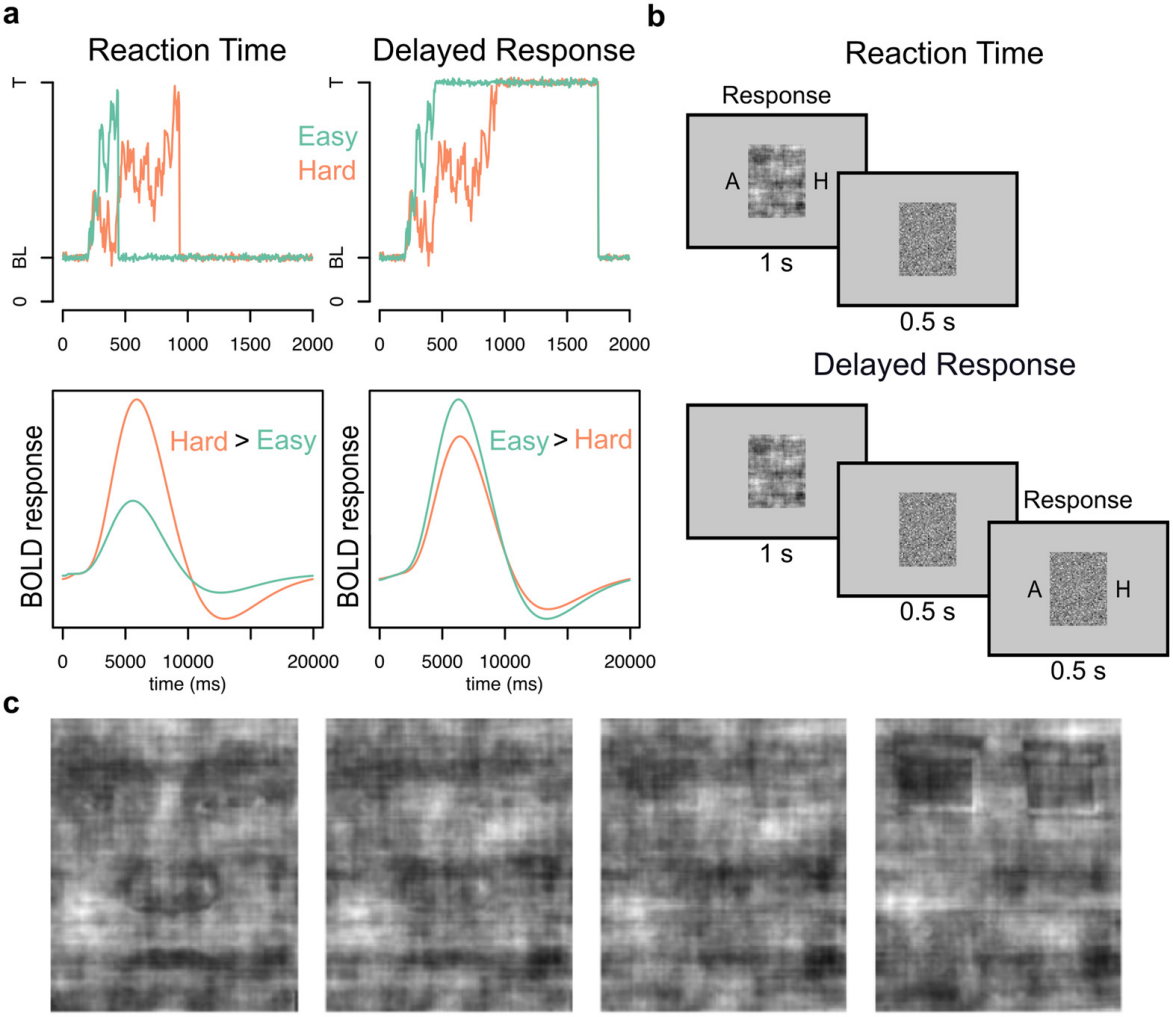
Blood oxygenation level dependent (BOLD) response predictions, experimental task and stimuli. (A) Predictions of BOLD-response of accumulator area. The top figures displays simulated firing rate from baseline (BL) to decision threshold (T) of neurons in lateral intraparietal area in monkeys during reaction time (left) and delayed response (right) conditions for easy (green) and hard (orange) choices. The bottom two figures in (A) display expected BOLD response for these conditions, which were estimated by convolving the simulated firing rate with the canonical hemodynamic response function (HRF). (B) Task paradigm. Responses were given while viewing stimuli in the reaction time condition (top), while after a forced delay in the delayed response-condition. The presentation of the letters “A” and “H” (representing the words face (“Ansikt”) and house (“Hus”) in Norwegian) informed participants that a response could be given. (C) Images of faces and houses were divided in the following categories depending on level of phase coherence and stimulus type (from left): easy face, hard face, hard house and easy house.

This study was designed to investigate which of two alternative decision and response-mechanisms is implemented during human perceptual decision making. Either the same neuron-populations/brain regions accumulate evidence and maintain the response like in monkeys, or evidence accumulation and maintenance of decisions are implemented in different brain regions. To formulate hypotheses for the comparison of these two alternatives, we derived predictions for blood oxygenation level dependent (BOLD) responses for a combined evidence accumulation and choice maintenance region during hard and easy decisions, by convolving the hypothesized firing rates of decision neurons with the canonical (double gamma) hemodynamic response function [16,17]. Fig 1A shows that a brain region with such neurons should show a response mode by difficulty crossover interaction so that the BOLD response is greater for hard than easy decisions when responses are given as soon as the decision boundary is reached, but greater for easy than hard decisions when choices are maintained and responses are given after a delayed response cue. In contrast, if evidence accumulation and choice maintenance are dissociated in humans and accumulation neurons stop firing when the decision boundary is reached, accumulator regions would be expected to be activated more for hard than easy decisions across response modes, and independent choice maintenance regions should show greater activation during delayed choice.

## Materials and Methods

### Ethics Statement

The study was approved by the ethics committee of the Department of Psychology at the University of Oslo and was conducted at the University of Oslo (Oslo University Hospital). All participants gave written informed consent.

### Participants

Twenty healthy participants (10 females) in the age range 23–40 (M = 29.36; SD = 6.16) took part in the study. All were right-handed and had normal or corrected-to-normal vision, and were paid 200 NOK to participate. Data from two participants were excluded from the analysis; one due to technical difficulties, while another participant had a strong bias towards responding in favor of one of the alternatives, which resulted in overall accuracy and response times not comparable to the other participants, leaving 18 participants (10 females). Of the participants included in the analysis, one experienced technical difficulties on one of three runs (see Procedure below).

### Design

A two-alternative forced choice perceptual task with face and house stimuli was used. The design was a 2*2 factorial design with response condition and difficulty level as independent variables. The dependent variables were accuracy, response times and BOLD response.

### Stimulus preparation

Images were taken from a pool of 25 face images (face database, Max Planck Institute for Biological Cybernetics, http://faces.kyb.tuebingen.mpg.de/) and 25 house images (provided by Flavia Filimon) that were 131*156 pixels large, and subtended 5° degrees visual angle horizontally. A varying degree of noise was added to the images to manipulate difficulty. All images were equated for spatial frequency, luminance, and contrast. They all had identical magnitude spectra, and their phase spectra were manipulated by using the weighted mean phase [18] technique to generate a set of images characterized by their percent of phase coherence. Four stimuli were created from each image by adding different levels of noise, resulting in 100 unique face images and 100 unique house images. The four difficulty levels were grouped into two for the fMRI-analysis (see below) to increase power in detecting differences: easy and difficult (Fig 1C), with different coherence levels for faces and houses (easy house: 51% and 54% coherence; hard house: 43.7% and 46.5% coherence; easy face: 50% and 53% coherence; hard face: 42.7% and 44.5% coherence). The differences in coherence levels for the same difficulty level across stimulus type were used to better align accuracy of responses for house and face stimuli. Later, when referring to difficulty levels for both face and house stimuli, we will use the coherence levels for face stimuli, and report coherence in proportion coherence.

### Behavioral task

Participants performed a two-alternative forced-choice perceptual task, with face and house stimuli. There were two response conditions (Fig 1B): the reaction time (RT) condition and the delayed response (DR) condition, performed in separate runs. All stimuli were presented on a grey (RGB values: 127, 127, 127) background. Trials in the two conditions were identical up to the presentation of the target stimulus. Using a jittered event-related design, each trial started with the presentation of a white (RGB: 255, 255, 255) fixation cross, which was displayed between 2 and 9 seconds. A red (RGB: 255, 0, 0) fixation cross followed for 0.5 seconds, to cue the participant to the upcoming task, followed by a scrambled image for another 0.5 seconds. The scrambled images were created by randomly scrambling tiles of 2 by 2 pixels from each of the 200 target stimuli. Scrambled images were included to provide a baseline for pupillometry-analysis of eye-tracking data. (The eye-tracking data were not used in the analysis due to the low quality of eye-tracking data we obtained in the scanner.) The target image was presented after the scrambled image. The target stimulus in each trial was chosen in a pseudo-random fashion for each participant, where it was made sure that a close to equal amount of face and house stimuli was presented in each run. In the RT-condition, participants responded during the 1-second presentation of the target stimulus. Responses were given using left or right index finger, and the letters A (for “ansikt”, face in Norwegian) and H (for “hus”, house in Norwegian) were shown on each side of the stimuli to indicate which index finger to use to respond face or house (counterbalanced across participants). After presentation of the target stimuli, the same scrambled image was presented for 0.5 seconds. In the DR-condition, the target stimulus, without the letters flanking the stimuli, was presented for the same duration as in the RT condition (1 second). Differently from the RT-condition, the offset of the target stimulus was followed by a cued delay period of 500 ms where responses were not allowed, and during which the scrambled image was presented. The delay duration was set to 500 ms so as to both be long enough to detect the hypothesized interaction, but not so long that the duration of the maintenance would make it impossible to detect difficulty effects. After the cued delay, the letters A and H were presented on each side of the scrambled images. Participants had been instructed beforehand to use the appearance of the letters as a cue to respond. Responses could be given in a 500 ms time window where the scrambled image was presented together with the letters. The letters were removed once a response was given, to indicate that the response was registered.

While in the scanner, MR-compatible response grips were used to obtain responses (ResponseGrip^®^, NordicNeuroLab, Bergen, Norway), and the stimuli were presented using eye-tracking goggles with two LCD-displays (VisualSystems^®^, NordicNeuroLab, Bergen, Norway), both with a screen resolution of 800*600 pixels and refresh rate of 85 Hz. During training outside the scanner (see Procedure below), stimuli were presented on a Dell laptop with a 15.6 inch screen, 1920*1080 pixels resolution and 60 Hz refresh rate, using keyboard buttons to respond. The Presentation^®^ software (Version 14.9, www.neurobs.com) was used to control the stimulus display and record responses.

### Procedure

Prior to entering the scanner participants performed two runs of the RT condition and one run of the DR condition in a training session, each run consisting of 70 trials. The training was performed in a quiet room. The training session was used to prevent strong learning effects while in the scanner. Information was given about response condition and which buttons to press for each response prior to each run in both training and main experiment. The main experiment consisted of three runs, each 112 trials long. The first and last runs were in the RT condition, and the middle run was in the DR condition. Each run lasted about 14 minutes and 17 seconds in the scanner. Behavioral data from the RT condition were analyzed with the drift diffusion model, a type of sequential sampling model (see Drift Diffusion Modeling section below). This model assumes choices are made when a decision boundary is reached, and thus could not be used to analyze results from the DR condition, where responses were made after a cued delay. To improve the reliability of the drift diffusion model analyses, we chose to have twice as many RT trials, with two runs in the RT condition and only one run in the DR condition.

An important precondition for the validity predictions for a combined accumulation-maintenance region is that participants had generally completed the accumulation phase of the decision process before onset of the delay-cues after 1s. We therefore used the fact the participants learn to adapt their response time to the available time and let all participants start with the RT condition, where the maximally allowed response time was 1 second (from stimulus onset to off-set of the response cues). All reported results are based on data from both RT-runs, except for one participant who, due to technical difficulties, did not complete the second RT-run. To verify that our results do not depend on a larger number of RT- than DR-trials, we also performed an analysis with matched number of trials from each condition by using only the first run from the RT condition. The results from this analysis were consistent with the analysis using both runs from the RT condition. As the analysis was consistent using one or two runs from the RT condition, the participant with only one complete RT-run is included in the reported results.

### Bayesian inference for behavioral data

We used a Bayesian approach to estimate accuracy and response time across difficulty levels and conditions[19]. Posterior distributions of accuracy and response time parameters were estimated with jags, a program to sample from posterior distributions in Bayesian data analysis using the gibbs sampler [20]. We used the rjags package [21] to interface with jags in the statistical programming language R [22].

We modeled response times as following a gamma distribution[23] while accuracy was assumed to follow a beta distribution. Variances in both accuracy and response time were drawn from gamma distributions. Non-informative priors were used to estimate group level posterior distributions. All gamma distributions were parameterized by shape and rate parameters that were obtained by transforming mean and standard deviations drawn from uniform distributions (0.01,30). Priors for α and β parameters on group level beta distributions were both set to 1, resulting in a non-informative group level beta distribution.

Mean response times were estimated separately for the RT and DR conditions with a hierarchical model (see S1 Fig for graphical depiction of model) containing following parameters:

1. For each level of coherence, one group level gamma distribution for the means of the individual level gamma distributions for mean response times.
2. For each level of coherence, one group level gamma distribution for the variances of the individual level gamma distributions for response times variances.
3. For each level of coherence, 18 individual level gamma distributions for the mean of the gamma likelihood function.
4. For each level of coherence, 18 individual level gamma distributions for the variance of the gamma likelihood function, so that response time likelihoods were estimated as:
  $$response\_time_{c,j,i}\;\sim \;gamma\left(\mu_{cj}{}^{2}/\;\sigma_{c,j}{}^{2},\;\mu_{c,j}\;/\;\sigma_{c,j}{}^{2}\right),$$ (1)
  where *μ* and *σ* are group level distributions, *c* indexes coherence, *j* participant, and *i* trial

Mean accuracies were estimated separately for the RT and DR condition with a hierarchical model (see S2 Fig for graphical depiction of model) containing following parameters:

1. For each level of coherence, one group level beta distribution for the means of the individual level beta distributions for mean accuracy.
2. For each level of coherence, one group level gamma distribution for the variance of the individual level beta distributions.
3. For each level of coherence, 18 individual level beta distributions for the mean of the binomial likelihood function:
  $$z_{c,j}\;\sim \;binomial\left(theta_{c,j},N\right),$$ (2)
  where *z* equals number of correct responses within coherence level *c* for participant *j* and *N* represents number of trials within coherence level *c* for participant *j*.

Values sampled from the posterior distribution for group mean parameters at each difficulty level for accuracy and response times are reported. Chain convergence was assessed by Gelman and Rubin’s $\hat{R}$ convergence diagnostic [24]. Measures of differences of posterior distributions were calculated by subtracting posterior distributions of one parameter from another on a sample by sample basis. When several parameters were combined, for example when grouping the four coherence levels into easy and hard, this was done by computing a grouped chain as the sample by sample mean of the constituent chains [25]. Differently than in classical null hypothesis testing, there is no unique convention to report results or to determine what a statistical significant result is. Following [19] we report the results of comparisons between conditions or coherence levels by stating the proportion of posterior samples of the difference between conditions that are above zero. This value can be understood as the posterior probability that the difference between two conditions or coherence level is larger than zero. In addition, we report 95% highest density intervals as a measure of uncertainty.

### Drift Diffusion Model

Behavioral results from the RT condition were analyzed with the drift diffusion model using the python toolbox HDDM (version 0.5) [26]. HDDM allows hierarchical Bayesian parameter estimation of the drift diffusion model, which uses trial-by-trial response time and accuracy data to estimate parameters describing how different aspects of simple two-choice decisions are expressed. In particular, the drift diffusion model assumes that a decision process begins at a starting point (parameter *z*) that lies between two decision boundaries whose distance is captured by a boundary separation parameter (*a*). It is assumed that during the decision process noisy evidence for the two response options (here face and house) is subtracted, and that this difference signal accumulates until one of the decision boundaries is reached. The speed of this accumulation process is measured by a drift rate parameter (*v*). The three main parameters of the drift diffusion model, *z*, *a*, and *v*, capture participants response bias, speed-accuracy trade-off, and the task difficulty, respectively. In addition, parameters for non-decision time and between trial variation of non-decision time, drift rate and boundary separation can capture additional response time effects.

### fMRI Data Acquisition

A 3 Tesla Philips Achieva whole body MR scanner was used for fMRI data acquisition, with an 8-channel Philips SENSE head coil (Philips Medical Systems, Best, the Netherlands). A T2* echo-planar imaging sequence (repetition time (TR), 2250 ms; echo time (TE), 30 ms; FOV, 240*240*114; flip angle, 80°; interleaved acquisition) with 38 slices and a voxel size of 3*3*3 mm were taken while participants performed the task. One scanning session consisted of 381 volumes, taking approximately 14 minutes and 17 seconds. An additional 5 dummy scans were taken before the experiment started to allow the MR signal to reach equilibrium. Anatomical T1 images with 170 slices and a voxel size of 1*1*1mm were recorded for registration of the functional images (TR, 6.6 ms; TE, 3.1 ms; FOV 256*256, flip angle, 8°).

### fMRI Analysis

Data were analyzed using a mixed effects general linear model in FSL [27]. The following preprocessing steps were taken: Motion correction using FMRIB's Linear Image Registration Tool (MCFLIRT), brain extraction using the Brain Extraction Tool (BET) function, spatial smoothing (with a Gaussian kernel of 5 mm full-width at half maximum), high-pass temporal filtering (>100 seconds) and slice timing correction. The design matrix of the General Linear Model (GLM) contained 8 explanatory variables of interest plus motion correction parameters and missed trials (4% of all trials) as nuisance variables. The explanatory variables (EV) of interest were separated into correct and incorrect decisions for easy faces, easy houses, hard faces and hard houses. Stimulus duration was set to the response time (i.e. from onset of target stimulus until response) for each trial, including the delay period in the DR condition. The four explanatory variables containing error trials (7.5% of all trials) were not included in the reported contrasts. Each subject’s individual run was analyzed with a first-level analysis. Then, a second level analysis with fixed effects was performed to combine the three runs within participants. Contrasts were created, separately for the RT and DR runs, in the second level analysis to compare differences in activation between difficulty levels and stimuli. Finally, a group level analysis combining the second level analysis from each subject was run using FMRIB’s local analysis of mixed effect (FLAME 1+2) with robust outlier detection. Z statistics images were cluster-threshold at Z > 2.3. Clusters with p<0.05 after correction for multiple comparisons (familywise error) in the regions of interest were reported as significant activations. In addition to a whole-brain analysis, we ran a region of interest analyses to identify activations in previously reported accumulator regions. The regions that were cluster corrected included the IPS, left dlPFC, right insula, left inferior frontal sulcus and dmPFC. Clusters larger than 59 voxels, as determined with the AFNI 3dClustSim tool to equal a family wise alpha of 0.05, surviving a threshold of Z>2.3 were deemed significant. The procedure described for the main fMRI analysis was also performed on an analysis on the RT runs, where explanatory variables for all face and all house trials (including error trials) were weighted with individual estimates of drift rate.

### Reverse inference meta-analysis

To infer which cognitive function were most likely involved in the different conditions, given observed activation patterns, we conducted a formal reverse inference meta-analysis [28] This meta-analysis quantifies the association between brain activation and terms describing perceptual, emotional, cognitive, and motor functions. These terms are single- or two-word combinations that authors used in their articles, and can thus be assumed to describe the function investigated in an experiment. Our meta-analysis used the tools in the Neurosynth package, but extended the underlying list of terms and activation location databases. We extended the list of terms because (a) the original Neurosynth list contains only single-word terms, whereas two-word terms are often more informative; (b) the Neurosynth database treats different forms of the same word (e.g., plural and singular, past and present forms) as different terms, whereas we used word-stems to avoid this; and (c) the Neurosynth word list is sourced from word frequencies in articles without systematic consideration of the accumulated knowledge about types of (cognitive) functions, whereas we extended this body of knowledge by adding terms from the Cognitive Atlas [29].

To better reflect the current literature, we expanded the dataset used for our meta-analysis by including activation locations stored in the BrainMap database [30]. Because articles are manually entered in this database, it contains more specific data (i.e., clear descriptions of contrasts associated with locations) than the Neurosynth database. On the other hand, it contains data from fewer articles (2,390 in BrainMap vs. 5,900 in Neurosynth) and allows meta-analyses only for relative broad areas of functioning. Combining the locations from the BrainMap and Neurosynth databases resulted in a new location database with locations from 7,500 unique articles (i.e., an increase of about 25% relative to the original Neurosynth database).

Expanding the Neurosynth location database necessitated extraction of terms mentioned in all papers in a consistent manner. Two general approaches can be used to distinguish relevant terms (i.e., those describing the topic of an article) from irrelevant ones. First, one can check the frequency of every word used in an article and define relevant words as those exceeding a threshold (Neurosynth uses 0.1%). Second, one can assume that all words in title, abstract, and keywords (except stop words, see below) describe the topic of an article, so that the occurrence of a term in these fields indicates that the paper indeed investigated the function described by that term. As we see no strong arguments to prefer either method, and because the second method is faster to implement (i.e., in most cases, it requires only access to PubMed, whereas the first requires full text access to all articles), we used the second method.

To calculate posterior probabilities of terms given the observed activations as described by Yarkoni and colleagues [28], we conducted the following steps:

1. As described above, we generated a list of terms describing cognitive and affective processes by (a) starting with the terms used in the Neurosynth database (https://github.com/neurosynth/neurosynth-data/blob/master/features.txt), (b) adding one- and two-word terms found in the Cognitive Atlas (http://www.cognitiveatlas.org/concepts/a), and (c) stemming all words in the term list and removing stop words (e.g., “of,” “by,” “end”) with the natural language toolkit (http://nltk.org).
2. We compiled a database with activation locations indexed by PubMed IDs for all articles in the Neurosynth (https://github.com/neurosynth/neurosynth-data/blob/master/features.txt, retrieved November 15^th^ 2013) and in the BrainMap databases (http://www.brainmap.org, retrieved with “Sleuth” on November 15^th^ 2013). This new, combined database comprises activation locations for 7,500 articles.
3. We compiled a new feature database by retrieving title, abstract, and keywords for each article, concatenating these strings, stemming and removing stop words, and testing for occurrence of terms from our term list in the concatenated string (i.e., at least one occurrence in either the title, abstract, or keywords of an article). All terms that occurred in fewer than 15 articles as well as the redundant or overly general terms “face*”, “house”, “picture”, “actor” were removed from the resulting database.
4. We used the Neurosynth toolbox (https://github.com/neurosynth/) to calculate the posterior probability of a term given activation at a location.

The posterior probability is defined as

$$\text{p}\left(\text{Term}|\text{Actv}.\right)=\frac{\text{p}\left(\text{Actv}.|\text{Term}\right)*\text{p}\left(\text{Term}\right)}{\text{p}\left(\text{Actv}.|\text{Term}\right)*\text{p}\left(\text{Term}\right)+\text{p}\left(\text{Actv}.|\text{notTerm}\right)*\left(1-\text{p}\left(\text{Term}\right)\right)}.$$ (3)

Using the posterior probability to select terms ensures that only those terms are selected that are consistently associated with activation at a given location and that at the same time this location is rarely activated in articles not mentioning the term. Put differently, focusing on high posterior probabilities focuses the interpretation of activation on terms with high specificities.

Because cognitive processes are often implemented in a distributed manner and multiple processes can influence decision making in our task, we identified multiple peak locations for each contrast. Peak locations and associated terms were identified as follows:

- Within each cluster, we identified local maxima using FSL’s cluster command (min distance between local maxima: 3cm).
- For each location (local maximum in a cluster), we created a region of interest (ROI) as a sphere with 5 mm radius around the location and calculated the average posterior z-value for each term for the ROI. Specifically, posterior z-values were calculated within the Neurosynth meta-analysis from chi-square statistics on posterior probabilities, such that the number of articles mentioning a term as well as the posterior probability influences the z statistic. Average posterior z-values for a term and ROI were calculated as weighted means of posterior z-values, using the z-statistic of our underlying fMRI contrast (normalized so that they summed to 1) as weights.
- To extract the most relevant cognitive terms, we calculated an “evidence score” by multiplying the peak z-value from our fMRI contrast in each ROI with the average z-value for the posterior probability of each term in same ROI. This multiplicative approach insures identification of terms associated with locations with strong activation, and for which the posterior probability given an activation location is high (by comparison, an additive approach could highlight terms that fulfill only one of the two conditions). We then extracted for each contrast the eight terms with the highest evidence score. When contrasts had only a single cluster, we extracted for each cluster the three terms with the highest evidence score.
- If a term was associated with multiple peak locations within a contrast, only the highest z-value for this term was extracted.

This procedure resulted in a list of terms that have a high posterior probability given the contrast image and can be considered to provide an unbiased/data-driven picture of the cognitive processes associated with a contrast.

## Results

We collected fMRI-data from participants performing a face-house discrimination task with four difficulty levels, implemented by manipulating the phase coherence of images (Fig 1C), in two response conditions. In the reaction time (RT) condition participants responded during stimulus-presentation. In the delayed response condition (DR) participants responded when a response cue appeared after a forced delay (Fig 1B).

### Behavioral results

**Fig 2:**
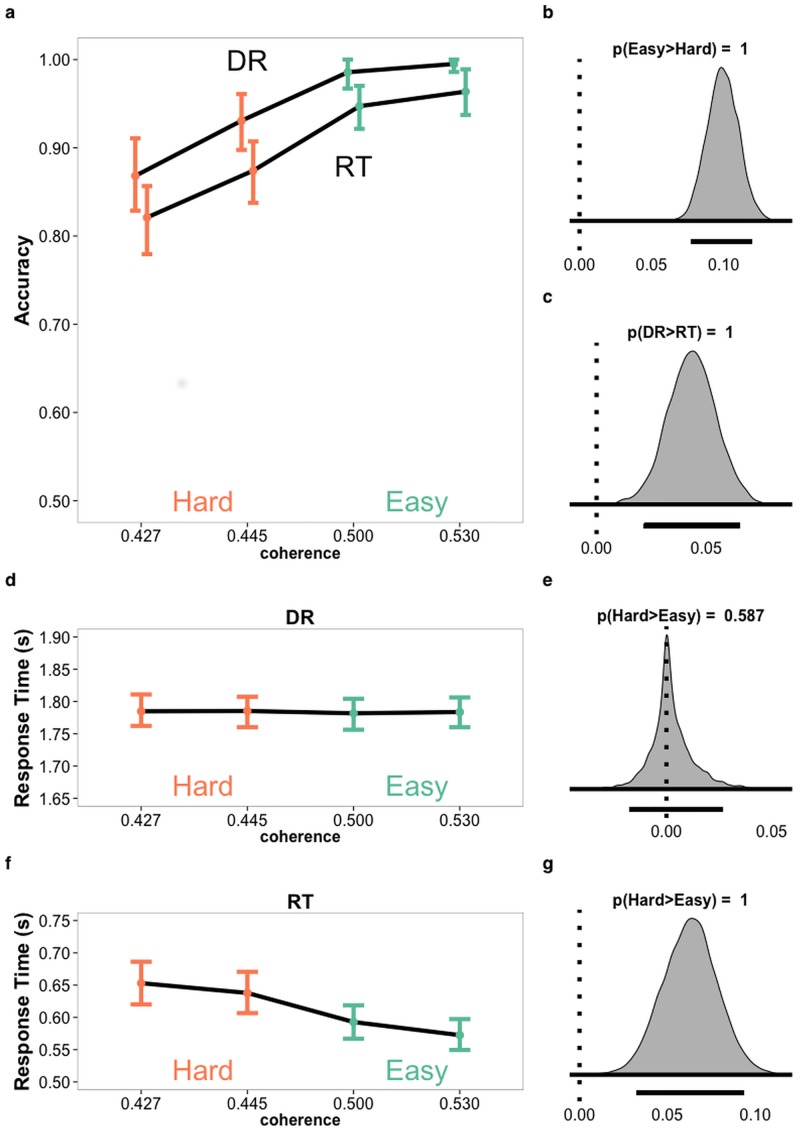
Posterior distributions of behavioral results. Estimated posterior distributions for accuracy (A) and response time in delayed response (DR) (D) and reaction time (RT) (F) conditions by coherence levels with error bars representing the 95% highest density intervals (HDI). Density plots for chains of difference in posterior distributions grouped to easy (green) and hard (orange) for accuracy (B) and response time in DR (E) and RT (G). Difference in combined chains of posterior distributions for accuracy in DR and RT (C). Line below density plots represent 95% HDI of chains of difference.

As expected, a Bayesian analysis of participants’ responses showed that accuracy increased with stimulus quality (p(accuracy|easy > accuracy|hard) = 1) (Fig 2A and 2B) and was generally higher in the DR condition (p(accuracy|DR > accuracy|RT) = 1; Fig 2A and 2C). In the RT condition, harder trials lead to slower responses (p(response time|hard > response time|easy) = 1) (Fig 2F and 2G), whereas response time was approximately the same for easy and hard in the DR condition (p(response time|hard > response time|easy) = .587; Fig 2D and 2E), suggesting that the accumulation process in the DR condition was generally completed before onset of the response cue. According to Gelman and Rubin’s $\hat{R}$ convergence diagnostic [24], the chains successfully converged, with $\hat{R}$ values for all parameter estimates between 1 and 1.02.

### Drift Diffusion Model results

**Fig 3:**
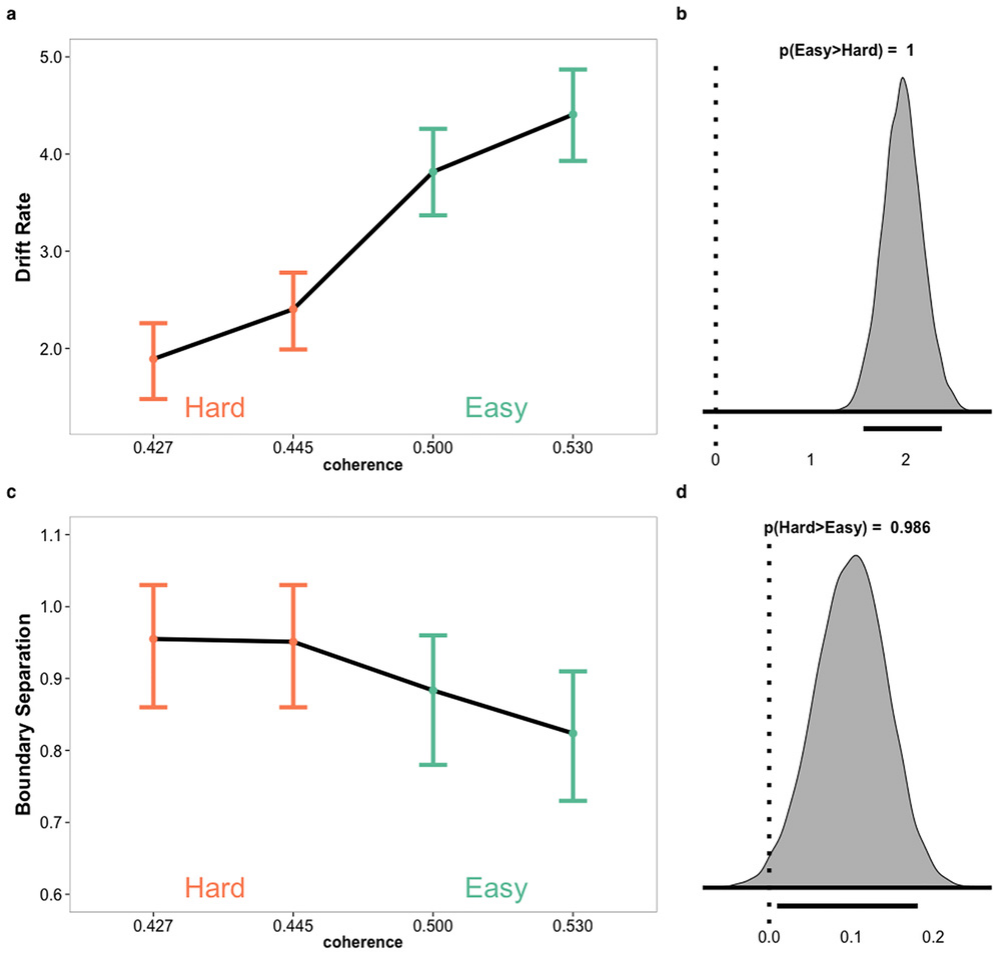
Posterior distributions of drift diffusion model parameters. Estimated posterior distributions for drift rate (A) and boundary separation (C) by coherence levels with error bars representing the 95% highest density intervals (HDI). Density plots for chains of difference in posterior distributions grouped to easy (green) and hard (orange) for drift rate (B) and boundary separation (D). Line below density plots represent 95% HDI of chains of difference.

To further test that the behavioral data were consistent with a sequential sampling account of decision making, we fitted results from the RT condition with a hierarchical Bayesian implementation of the drift diffusion model [2,26]. The model we used estimated individual and group parameters for drift rate (v), boundary separation (a) and non-decision time (t), as well as group estimates for inter-trial variability of both drift rate and non-decision time. Drift rate and boundary separation were estimated for each difficulty level. We allowed the boundary separation to vary as a function of task difficulty, even though task difficulty has the strongest influence on the drift rate. We chose this approach because it is a well-known phenomenon that decision makers respond to increased task difficulty by applying a more conservative decision criterion [31]. While our stimulus presentation time was relatively short, it was still long enough for participants to realize if the trial was easy or hard, and to thus adjust their decision criterion. As is typically observed in tasks with varying difficulty levels [31], we found that higher coherence levels led to higher drift rate (p(drift rate|easy > drift rate|hard) = 1) (Fig 3A and 3B) and lower boundary separation (p(boundary|hard > boundary|easy) = .986; Fig 3C and 3D, see S1 Table for mean and distribution measures of individual and group parameter estimates and S3 Fig for plots of individual means of parameter estimates). A posterior predictive check indicated that the parameter estimations were able to replicate the observed RT-distributions for correct and error-responses (see S4 Fig for all posterior plots). Model fit was estimated with the Deviance Information Criterion (DIC). Lower DIC values indicate better fit. The chosen model had a good fit (DIC: -4908), compared to a model that also included inter-trial variability of drift rate and non-decision time, but ignored difficulty (DIC: -4399). It also outperformed models that were identical to the chosen model, but where only drift rate (DIC: -4896) or boundary separation (DIC: -4608) changed across difficulty levels. The $\hat{R}$ values for all parameter estimates were between 1 and 1.006, indicating convergence.

### fMRI results

**Fig 4:**
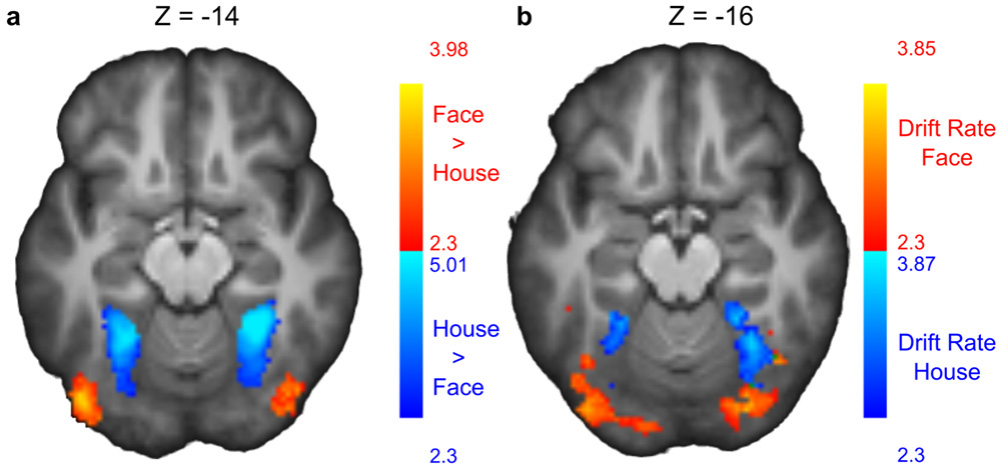
Face and House cluster activations. (A) Cluster activations for face>house (red) and house>face (blue) contrasts collapsed across response conditions. (B) Clusters positively correlated with drift rates estimated for each subject across difficulty levels. Green indicates overlap of clusters. All contrasts were thresholded at Z>2.3 with whole-brain correction for multiple comparisons at p<0.05.

**Fig 5:**
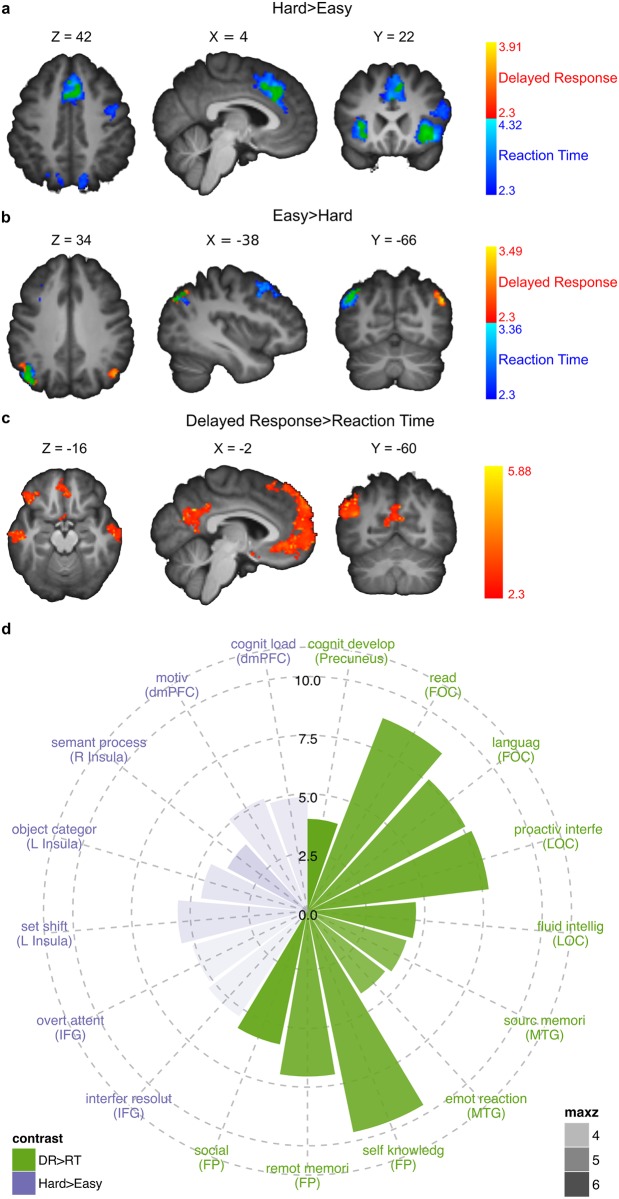
Cluster activations and polar plot. Cluster activations for hard>easy (A) and easy>hard (B) contrasts for reaction time (RT) (blue) and delayed response (DR) (red) with overlapping activations in green. Threshold at Z>2.3, cluster corrected to p<0.05 with 3dClustSim. (C) Cluster activations for DR>RT contrast, whole-brain corrected for multiple comparisons at p<0.05 with cluster threshold at Z>2.3. (D) Posterior probabilities of terms describing psychological functions. In purple are terms with high posterior probability from regions activated for hard>easy across RT and DR conditions. In green are terms from DR>RT contrast. Posterior probabilities were converted to z values and are shown on the radial axis. Transparency represents the z value of the contrasts at each peak location. dmPFC = dorsomedial prefrontal cortex, FOC = frontal orbital cortex, FP = frontal pole, IFG = inferior frontal gyrus, LOC = lateral occipital cortex, MTG = middle temporal gyrus.

Confirming previous findings [12,32], activity in fusiform face area (Left: MNI coordinates X = -38, Y = -86, Z = -12, max z: 3.98, Right: 44, -72, -10, max z: 3.69) and parahippocampal place area (Left: -26, -50, -12, max z: 5.11, Right: 30, -48, -12, max z: 5.32) was correlated with the amount of evidence for face and house stimuli respectively (Fig 4A, see Table 1 for MNI coordinates and max z-values of all clusters from reported fMRI-contrasts). Activity in face (Left: -38, -82, -16, max z: 3.38, Right: 24, -86, -12, max z: 3.6) and house regions (Left: -24, -46, -16, max z: 3.35, Right: 26, -42, -18, max z: 3.87) also correlated with each subject’s individual estimates of drift rate across coherence levels (Fig 4B). Consistent with the prediction of activation in a combined accumulation and maintenance region, in the RT-condition (Fig 1A, left) the previously reported accumulator regions IPS (Left: -24, -66, 32, max z: 3.84, Right: 12, -72, 38, max z: 3.65), dmPFC (6, 26, 38, max z: 4.07), left inferior frontal gyrus (IFG) (-42, 4, 33, max z: 3.5) and right insula (34, 26, -6, max z: 4.32) were more strongly activated for more difficult decisions (Fig 5A), in addition to other regions (Table 1 **)**. Contrary to the prediction for a combined accumulation and maintenance area, none of the regions more strongly activated for hard than easy decisions in the RT-condition were significantly activated more for easy than hard decisions in the DR-condition (Fig 1A, right and Fig 5B). We also performed an interaction analysis to check if any regions showed the hypothesized interaction based on firing rates in the monkey LIP. Three regions were significantly activated, however, these did not follow the requirements of being more activated for hard than easy decisions in the RT condition and more for easy than hard decisions in the DR condition (S5 Fig displays signal changes for these three regions across difficulty levels and condition). Hence, no brain region displayed an interaction in activation that was consistent with the neural firing pattern observed in monkey accumulation neurons. Instead, the following regions were activated more for hard than easy decisions also in the DR condition: dmPFC (0, 34, 36, max z: 3.18), bilateral insula (Left: -30, 22, -8, max z: 3.11, Right: 34, 24, -6, max z: 3.91), and right IFG (40, 8, 24, max z: 3.16) (Fig 5A). A greater BOLD response for hard decisions was also identified in the IPS and left IFG, but these did not reach significance (left IFG: -36, 10, 30, max z: 3.13, p>0.05, left IPS: -18, -68, 34, max z: 2.81, p>0.05). Lastly, using individual estimates of drift rate, we found that the regions more activated for hard than easy decisions in both the RT and DR conditions were also negatively correlated with drift rate in the RT condition, which we hypothesized would be a proxy for identifying an accumulation region (Table 1, S6 Fig).

**Table 1:** Areas with significant activation, in MNI coordinates.

Contrast	Area	Hemi	voxels	max z-score	Peak voxel		
					X	Y	Z
**RT**							
Hard>Easy							
	Insula and Inferior Frontal Gyrus	R	1970	4.32	34	26	-6
	Dorsomedial Prefrontal Cortex	R	1598	4.07	6	26	38
	Insula	L	763	4.32	-32	24	2
	Inferior Frontal Gyrus	L	320	3.5	-42	4	32
	Intra Parietal Sulcus	L	213	3.84	-24	-66	32
	Middle Frontal Gyrus	R	171	3.14	38	0	46
	Intra Parietal Sulcus	R	138	3.65	12	-72	38
	Cuneal Cortex	L	78	3.07	-8	-80	38
	Lateral Occipital Cortex	R	78	3.44	32	-66	24
Easy>Hard							
	Superior/Middle Frontal Gyrus	L	604	3.36	-24	32	46
	Lateral Occipital Cortex	L	411	3.25	-46	-62	28
**DR**							
Hard>Easy							
	Dorsomedial Prefrontal Cortex		353	3.18	0	34	36
	Insula	R	270	3.91	34	24	-6
	Insula	L	169	3.11	-30	22	-8
	Inferior Frontal Gyrus	R	77	3.16	40	8	24
Easy>Hard							
	Lateral Occipital Cortex	L	269	3.31	-44	-66	36
	Occipital Pole	R	227	3.49	18	-90	16
	Lateral Occipital Cortex	R	162	3.43	50	-66	32
	Occipital Pole	L	64	3.08	-14	-90	24
**DR>RT**							
	Frontal Pole	L	4235	6.36	-4	58	20
	Temporal Gyrus	L	1067	5.7	-66	-14	-18
	Lateral Occipital Cortex	L	867	6.2	-44	-62	38
	Frontal Orbital Cortex	L	844	6.1	-48	36	-12
	Precuneus	L	709	6.54	-4	-58	30
	Middle Temporal Gyrus	R	609	5.6	62	-10	-26
**Face>House**							
	Lateral Occipital Cortex	L	511	3.98	-38	-86	-12
	Lateral Occipital Cortex	R	194	3.69	44	-72	-10
**House>Face**							
	Temporal Occipital Fusiform Cortex	R	701	5.32	30	-48	-12
	Temporal Occipital Fusiform Cortex	L	520	5.11	-26	-50	-12
**Face Drift Rate**							
	Lateral Occipital Cortex	L	631	3.38	-38	-82	-16
	Occipital Fusiform Gyrus	R	362	3.6	24	-86	-12
	Temporal Occipital Fusiform Cortex	R	223	3.85	44	-50	-26
**House Drift Rate**							
	Temporal Occipital Fusiform Cortex	R	614	3.87	26	-42	-18
	Temporal Occipital Fusiform Cortex	L	229	3.35	-24	-46	-16
**Accumulation**							
	Medial Occipital Cortex	L	4444	4.05	-10	-80	6
	Dorsomedial Prefrontal Cortex	R	1533	4.02	8	18	40
	Frontal Orbital Cortex and Insula	R	913	4.14	38	24	-8
	Insula	L	530	3.66	-32	18	-10
	Inferior Frontal Gyrus	R	425	3.41	46	10	16

Contrasts under RT and DR header were cluster corrected to p<0.05 with 3dClustSim, while remaining contrasts were corrected for multiple comparisons at whole-brain level at p<0.05. All clusters were obtained by thresholding at Z>2.3. RT = reaction time; DR = delayed response; R = right; L = left; Hemi = hemisphere.

To identify regions involved specifically in decision maintenance we compared activation in the DR- and RT-conditions (DR>RT). The regions with greater activation during delayed responses were the frontal pole (-4,58,20, max z: 6.36), temporal gyri (Left: -66,-14,-18, max z: 5.7, Right: 62, -10, -26, max z: 5.6), left lateral occipital cortex (-44, -62, 38, max z: 6.2), left frontal orbital cortex (-48, 36, -12, max z: 6.1) and precuneus (-4, -58, 30, max z: 6.54) (Fig 5C, Table 1).

### Reverse inference meta-analysis

To further differentiate the potential roles of the regions activated more strongly for hard than easy decisions in both the RT- and DR-condition, while taking into account the difficulties associated with reverse inference from fMRI activations [33], we conducted a formal reverse inference meta-analysis based on the Neurosynth and BrainMap databases [28,34]. This analysis identifies the psychological functions with the highest posterior probability of being associated with activation in specific brain regions (Fig 5D). When using results of such an analysis it is important to acknowledge that their reliability relies on the soundness of the statistical approach and the quality of the underlying data. For reverse inference meta-analyses it is the quality of the data, which consist of broad cognitive terms associated with activation locations, that limits the strength of conclusions that can be drawn from the analysis, as the resolution of these data is low compared to the fine grained processes of cognition and decision making as described in for example the DDM. Hence, the results of such an analysis should be seen more as an interpretation aid, and should not be considered as definitive evidence in favor of one or another explanation.

For the regions with greater activation in hard than easy trials the reverse inference meta-analysis results (Fig 5D, left) show that the dmPFC indicates motivational processes and high cognitive load, the right IFG indicates attentional processes and interference resolution, and the bilateral insula indicates set shifting, object categorization and semantic processing. Because all identified regions were associated with terms that could indicate accumulation-like processes, specifically cognitive load for dmPFC, object categorization for insula and interference resolution for IFG, the reverse inference analysis approach did not allow us to further narrow down the potential accumulator regions.

We also performed a reverse inference meta-analysis on the clusters activated during the delay period in the DR condition to better understand how these brain regions might maintain choices and initiate a response. The regions with greater activation in the DR- compared to the RT-condition were associated with a number of terms centering round processes of language, memory, and self-reflection (Fig 5D, right). While a clear interpretation of these results is difficult, they could indicate that participants attempt to bridge the delay by verbalizing their response and/or a stronger activation of a default network (as indicated by the high evidence for self knowledge).

## Discussion

We tested whether the BOLD signal pattern during simple perceptual decision making in humans is consistent with firing patterns of LIP neurons in monkeys, which both accumulate evidence and maintain decisions. We predicted that, if such a region exists in humans, it would be activated more for hard than easy decisions in a self-paced condition while also activating more for easy than hard decisions in a forced delay condition. No region showed an activation pattern consistent with these predictions, thus suggesting a different decision-response mechanism in humans than the one observed in LIP neurons of monkeys. In accordance with our alternative model, the results indicated a separation of accumulation and maintenance processes. By comparing brain activation between difficulty levels and response modes, we found that evidence accumulation is likely implemented in dmPFC, IFG and/or insula while potential choice maintenance regions span the frontal, temporal and occipital cortices. A quantitative reverse inference meta-analysis suggested that response-maintenance might rely on a verbalization of the response within the frontal pole.

The process of perceptual decision making in monkeys has been described as a process of embodied cognition, where the regions transforming evidence are directly involved in performing the motor activity needed to make a response [35], and “to see and decide is, in effect, to plan a motor response” [36]. Interpreting our results together with results of other recent studies suggest that the process of decision making is not embodied to the same degree in humans, as the accumulator regions we and others report are not directly associated with sensorimotor processes. For example, support for a more abstract mechanism of evidence accumulation in humans comes from Filimon and colleagues [15], who showed that left IFG, and not sensorimotor regions like IPS, accumulated evidence when the preparation of motor response was disentangled from the perceptual decision. Further, activation patterns have been reported to be the same across motor response modalities, when comparing responses given with button presses and saccades [14,37], but see [38,39]. Interestingly, decision and motor processes were also disentangled during decision making in a recent study with monkeys [40], although both processes were localized within the LIP. Comparing results from perceptual and value-based decision making studies indicates that accumulator regions in humans also might be general across decision domains, as both IPS and dmPFC have been identified as evidence accumulators in reward-based [9,11] and perceptual [10,41] decision making tasks. However, only experiments that implement both tasks can give conclusive answers of the generality of accumulator regions.

Our results suggested a dissociation of accumulation and maintenance processes. The most likely candidate regions for evidence accumulation would therefore be activated more for hard than easy decisions across response conditions, as prolonged accumulation for hard decisions leads to increased neural activity. While to our knowledge no other study has compared activation patterns for delayed and immediate responses in one study, the same brain regions that we found have been reported to be more activated for low than high stimulus quality regions in both immediate [10,14,41] and delayed [11,12,16] response tasks. Another area frequently reported to be responsible for accumulation is the IPS [9–11,41]. Our results do not provide strong evidence in favor of or against the IPS as accumulator, given that the IPS was activated more for harder decisions in both conditions, but only significantly so in the RT-condition.

There are potential alternative explanations for the activations we report as responsible for evidence accumulation, including time-on-task and motor planning. The reason why we believe a time-on-task explanation of our results is unlikely is that the areas we suggest as accumulators are activated more for hard than easy tasks in both the RT and DR condition. Difficulty correlates with response time in the RT condition (Fig 2G), but this is not the case in the DR condition, where we found no evidence supporting difference in response time between hard and easy trials (Fig 2E). As for motor planning, we cannot completely exclude that the regions we report as accumulators are instead/also involved in motor processes. However, none of the terms with the highest posterior probability in the reverse inference meta-analysis indicated motor processes, and the regions we report are typically not associated with motor preparation. Lastly, the model based fMRI analysis found that the regions that showed greater activation for harder trials were also negatively correlated with trial-by-trial variations of drift rate on an individual level. That is, these regions are associated with a marker of individual’s information processing.

Given a dissociation between accumulation and maintenance of decisions, we tried to identify regions responsible for maintenance of decisions by contrasting activity in the DR- and RT-conditions. This contrast led to identifying clusters within the frontal pole, temporal gyri, lateral occipital and frontal orbital cortex and precuneus. Using the reverse inference meta-analysis for these activations we found that the frontal pole could be responsible for verbalizing the response during the delay phase. An alternative interpretation of the activation differences between DR and RT is that they mainly reflect reduced default network activation in the harder RT compared to the easier DR condition (signal change plots in S7 Fig). However, we suggest that the DR vs. RT contrast does not mainly reflect an effect of difficulty, because regions obtained from this contrast do not show a clear effect when comparing trials with hard and easy stimuli, which have greater effect on accuracy than response condition.

While our reverse inference analysis from the Neurosynth and BrainMap database give unbiased results, the possible strength of the conclusions drawn from a database analysis is limited by the quality of the underlying data. In particular, the Neurosynth imaging data are based on peak coordinates and not complete activation images, and do not include information about specific contrasts. The reported reverse inference, similar to approaches in other studies [42], can therefore be understood as a first exploration of the differences in cognitive processes that are driven by different brain activation patterns.

In summary, our computational modeling and fMRI results suggest independent processes of accumulation and maintenance of perceptual decisions in humans, in which evidence accumulation is likely implemented in dmPFC, IFG and/or insula while potential choice maintenance regions span the frontal, temporal and occipital cortices.

## Supporting Information

S1 Fig Bayesian graphical model of response time estimations. The graphical model, inspired by[25], describes the dependencies in the hierarchical Bayesian model used to estimate response times. The response time *y*_*cji*_ from coherence level *c*, participant *j* and trial *i* depend on the shape *s*_*cj*_ and rate *r*_*cj*_ parameters of a gamma distribution, which are transformed from mean and standard deviation parameters. Parameters for each subject and coherence depend on coherence parameters. Coherence parameters depend on group parameters, which depend on non-informative priors. The "~" symbol describes that values are drawn from the above distributions while the " = " symbol means that values have a deterministic dependency. The ellipsis "…" symbol describes a repeated dependency, i.e. parameters are estimated for multiple coherence levels.
(TIFF)

S2 Fig Bayesian graphical model of accuracy estimations. The graphical model, inspired by[25], describes the dependencies in the hierarchical Bayesian model used to estimate correct responses. Number of correct responses *z*_*cj*_ of *N*_*cj*_ total responses from coherence level *c* and participant *j* depend on the value of the θ_cj_ parameter in a binomial distribution. Parameters for each subject and coherence depend on condition parameters. Coherence parameters depend on group parameters, which depend on non-informative priors. The "~" symbol describes that values are drawn from a distribution while the " = " symbol means that values have a deterministic dependency. The ellipsis "…" symbol describes a repeated dependency, i.e. parameters are estimated for multiple coherence levels.
(TIFF)

S3 Fig Individual drift diffusion model estimates. Individual parameter estimates for drift rate, boundary separation and non-decision time across coherence levels (except non-decision time).
(TIFF)

S4 Fig Posterior predictive checks. Observed RT distributions (red) for error (coded with negative reaction times) and correct (coded with positive reaction times) responses and predicted response distributions (blue) for each subject based on estimated parameter values across difficulty level.
(TIFF)

S5 Fig Signal change interaction regions. Percent signal change compared to baseline in delayed response (DR) and reaction time (RT) conditions across easy (green) and hard (orange) difficulty levels in clusters identified in interaction analysis (Z>2.3, corrected to p<0.05 at whole-brain level).
(TIFF)

S6 Fig Cluster activations for drift rate and difficulty contrasts. (A) Clusters identified to be negatively correlated with individual estimates of drift rate (Z>2.3, corrected to p<0.05 at whole-brain level) compared with (B) cluster activations for hard>easy contrasts for reaction time (RT) (blue) and delayed response (DR) (red) with overlapping activations in green (Z>2.3, cluster corrected to p<0.05 with 3dClustSim).
(TIFF)

S7 Fig Signal change maintenance regions. Percent signal change compared to baseline in reaction time (RT) (blue) and delayed response (DR) (red) conditions in clusters activated more in the delayed response than reaction condition.
(TIFF)

S1 Table Mean and distribution measures of posterior distributions of parameter estimates in drift diffusion model. SD = Standard Deviation. q = quantile.
(DOCX)

## References

[1] Usher M, McClelland JL. The time course of perceptual choice: the leaky, competing accumulator model. Psychological Review. American Psychological Association; 2001;108: 550. 10.1037/0033-295x.108.3.550 11488378

[2] Ratcliff R, McKoon G. The diffusion decision model: Theory and data for two-choice decision tasks. Neural Comput. MIT Press; 2008;20: 873–922. 10.1162/neco.2008.12-06-420 PMC2474742 18085991

[3] Shadlen MN, Newsome WT. Neural basis of a perceptual decision in the parietal cortex (area LIP) of the rhesus monkey. Journal of Neurophysiology. Am Physiological Soc; 2001;86: 1916–1936. 10.1152/jn.2001.86.4.1916 11600651

[4] Roitman JD, Shadlen MN. Response of neurons in the lateral intraparietal area during a combined visual discrimination reaction time task. J Neurosci. Soc Neuroscience; 2002;22: 9475–9489. 10.1523/JNEUROSCI.22-21-09475.2002 PMC6758024 12417672

[5] Gold JI, Shadlen MN. Representation of a perceptual decision in developing oculomotor commands. Nature. Nature Publishing Group; 2000;404: 390–394. 10.1038/35006062 10746726

[6] Horwitz GD, Newsome WT. Target selection for saccadic eye movements: prelude activity in the superior colliculus during a direction-discrimination task. Journal of Neurophysiology. Am Physiological Soc; 2001;86: 2543–2558. 10.1152/jn.2001.86.5.2543 11698541

[7] Kim JN, Shadlen MN. Neural correlates of a decision in the dorsolateral prefrontal cortex of the macaque. Nat Neurosci. NATURE AMERICA; 1999;2: 176–185. 10.1038/5739 10195203

[8] Bracewell RM, Mazzoni P, Barash S, Andersen RA. Motor intention activity in the macaque's lateral intraparietal area. II. Changes of motor plan. Journal of Neurophysiology. 1996;76: 1457–1464. 8890266 10.1152/jn.1996.76.3.1457

[9] Basten U, Biele G, Heekeren HR, Fiebach CJ. How the brain integrates costs and benefits during decision making. Proc Natl Acad Sci USA. National Acad Sciences; 2010;107: 21767–21772. 10.1073/pnas.0908104107 PMC3003102 21118983

[10] Kayser AS, Buchsbaum BR, Erickson DT, D'Esposito M. The Functional Anatomy of a Perceptual Decision in the Human Brain. Journal of Neurophysiology. 2010;103: 1179–1194. 10.1152/jn.00364.2009 20032247 PMC2887630

[11] Hare TA, Schultz W, Camerer CF, O'Doherty JP, Rangel A. Transformation of stimulus value signals into motor commands during simple choice. Proc Natl Acad Sci USA. National Acad Sciences; 2011;108: 18120–18125. 10.1073/pnas.1109322108 PMC3207676 22006321

[12] Heekeren HR, Marrett S, Bandettini PA, Ungerleider LG. A general mechanism for perceptual decision-making in the human brain. Nature. Nature Publishing Group; 2004;431: 859–862. 10.1038/nature02966 15483614

[13] Green N, Biele GP, Heekeren HR. Changes in Neural Connectivity Underlie Decision Threshold Modulation for Reward Maximization. Journal of Neuroscience. 2012;32: 14942–14950. 10.1523/JNEUROSCI.0573-12.2012 23100417 PMC6704845

[14] Ho TC, Brown S, Serences JT. Domain General Mechanisms of Perceptual Decision Making in Human Cortex. Journal of Neuroscience. 2009;29: 8675–8687. 10.1523/JNEUROSCI.5984-08.2009 19587274 PMC2719543

[15] Filimon F, Philiastides MG, Nelson JD, Kloosterman NA, Heekeren HR. How Embodied Is Perceptual Decision Making? Evidence for Separate Processing of Perceptual and Motor Decisions. Journal of Neuroscience. 2013;33: 2121–2136. 10.1523/JNEUROSCI.2334-12.2013 23365248 PMC6619122

[16] Liu T, Pleskac TJ. Neural correlates of evidence accumulation in a perceptual decision task. Journal of Neurophysiology. 2011;106: 2383–2398. 10.1152/jn.00413.2011 21849612

[17] Mulder MJ, van Maanen L, Forstmann BU. Perceptual decision neurosciences—A model-based review. NEUROSCIENCE. 2014;277: 872–884. 10.1016/j.neuroscience.2014.07.031 25080159

[18] Dakin SC, Hess RF, Ledgeway T, Achtman RL. What causes non-monotonic tuning of fMRI response to noisy images? Current Biology. Cell Press; 2002;12: R476–R477. 10.1016/s0960-9822(02)00960-0 12176342

[19] Kruschke JK. Bayesian Estimation Supersedes the t Test. Journal of Experimental Psychology: General. 2012 10.1037/a0029146 22774788

[20] Plummer M. JAGS: A program for analysis of Bayesian graphical models using Gibbs sampling. 2003.

[21] Plummer M, Stukalov A, Plummer MM. Package “rjags.” update. 2013.

[22] Team RC. R: A language and environment for statistical computing. R Foundation for Statistical Computing. 2012. ISBN 3-900051-07-0. Available: http://www.R-project.org/. 2013.

[23] Palmer EM, Horowitz TS, Torralba A, Wolfe JM. What are the shapes of response time distributions in visual search? Journal of Experimental Psychology: Human Perception and Performance. 2011;37: 58–71. 10.1037/a0020747 21090905 PMC3062635

[24] Gelman A, Rubin DB. Inference from Iterative Simulation Using Multiple Sequences. Statistical Science. Institute of Mathematical Statistics; 1992;7: 457–472.

[25] Kruschke JK. Doing Bayesian Data Analysis. Academic Press; 2010 pp. 1–542.

[26] Wiecki TV, Sofer I, Frank MJ. HDDM: Hierarchical Bayesian estimation of the Drift-Diffusion Model in Python. Front Neuroinform. 2013;7: 14 10.3389/fninf.2013.00014 23935581 PMC3731670

[27] Jenkinson M, Beckmann CF, Behrens TEJ, Woolrich MW, Smith SM. FSL. NeuroImage. 2012;62: 782–790. 10.1016/j.neuroimage.2011.09.015 21979382

[28] Yarkoni T, Poldrack RA, Nichols TE, Van Essen DC, Wager TD. Large-scale automated synthesis of human functional neuroimaging data. Nat Meth. 2011;8: 665–670. 10.1038/nmeth.1635 PMC3146590 21706013

[29] Poldrack RA, Kittur A, Kalar D, Miller E, Seppa C, Gil Y, et al The cognitive atlas: toward a knowledge foundation for cognitive neuroscience. Front Neuroinform. Frontiers Media SA; 2011;5. 10.3389/fninf.2011.00017 PMC3167196 21922006

[30] Laird AR, Lancaster JL, Fox PT. BrainMap: the social evolution of a human brain mapping database. Neuroinformatics. 2005;3: 65–78. 15897617 10.1385/ni:3:1:065

[31] Lo C-C, Wang X-J. Cortico–basal ganglia circuit mechanism for a decision threshold in reaction time tasks. Nat Neurosci. 2006;9: 956–963. 10.1038/nn1722 16767089

[32] Tremel JJ, Wheeler ME. Content-specific evidence accumulation in inferior temporal cortex during perceptual decision-making. NeuroImage. 2015;109: 35–49. 10.1016/j.neuroimage.2014.12.072 25562821 PMC4340815

[33] Poldrack RA. Can cognitive processes be inferred from neuroimaging data? Trends in Cognitive Sciences. 2006;10: 59–63. 10.1016/j.tics.2005.12.004 16406760

[34] Fox PT, Laird AR, Fox SP, Fox PM, Uecker AM, Crank M, et al Brainmap taxonomy of experimental design: Description and evaluation. Hum Brain Mapp. 2005;25: 185–198. 10.1002/hbm.20141 15846810 PMC6871758

[35] Gold JI, Shadlen MN. The Neural Basis of Decision Making. Annu Rev Neurosci. 2007;30: 535–574. 10.1146/annurev.neuro.29.051605.113038 17600525

[36] Rorie AE, Newsome WT. A general mechanism for decision-making in the human brain? Trends in Cognitive Science. 2005;9. 10.1016/j.tics.2004.12.007 15668095

[37] Heekeren HR, Marrett S, Ruff DA, Bandettini PA, Ungerleider LG. Involvement of human left dorsolateral prefrontal cortex in perceptual decision making is independent of response modality. Proc Natl Acad Sci USA. National Acad Sciences; 2006;103: 10023–10028. 10.1073/pnas.0603949103 PMC1479865 16785427

[38] Tosoni A, Corbetta M, Calluso C, Committeri G, Pezzulo G, Romani GL, et al Decision and action planning signals in human posterior parietal cortex during delayed perceptual choices. European Journal of Neuroscience. 2014;39: 1370–1383. 10.1111/ejn.12511 24612482

[39] Tosoni A, Galati G, Romani GL, Corbetta M. Sensory-motor mechanisms in human parietal cortex underlie arbitrary visual decisions. Nat Neurosci. 2008;11: 1446–1453. 10.1038/nn.2221 18997791 PMC3861399

[40] Bennur S, Gold JI. Distinct Representations of a Perceptual Decision and the Associated Oculomotor Plan in the Monkey Lateral Intraparietal Area. Journal of Neuroscience. 2011;31: 913–921. 10.1523/JNEUROSCI.4417-10.2011 21248116 PMC3380543

[41] Erickson DT, Kayser AS. The neural representation of sensorimotor transformations in a human perceptual decision making network. NeuroImage. Elsevier Inc; 2013;79: 340–350. 10.1016/j.neuroimage.2013.04.085 23631989

[42] Helfinstein SM, Schonberg T, Congdon E, Karlsgodt KH, Mumford JA, Sabb FW, et al Predicting risky choices from brain activity patterns. Proceedings of the National Academy of Sciences. 2014;111: 2470–2475. 10.1073/pnas.1321728111 PMC3932884 24550270

