# Supplementary figures and images for: Evidence Accumulation and Choice Maintenance Are Dissociated in Human Perceptual Decision Making
Source: PLoS One. 2015 Oct 28;10(10):e0140361. doi: 10.1371/journal.pone.0140361 (PMC4624809; doi:10.1371/journal.pone.0140361)

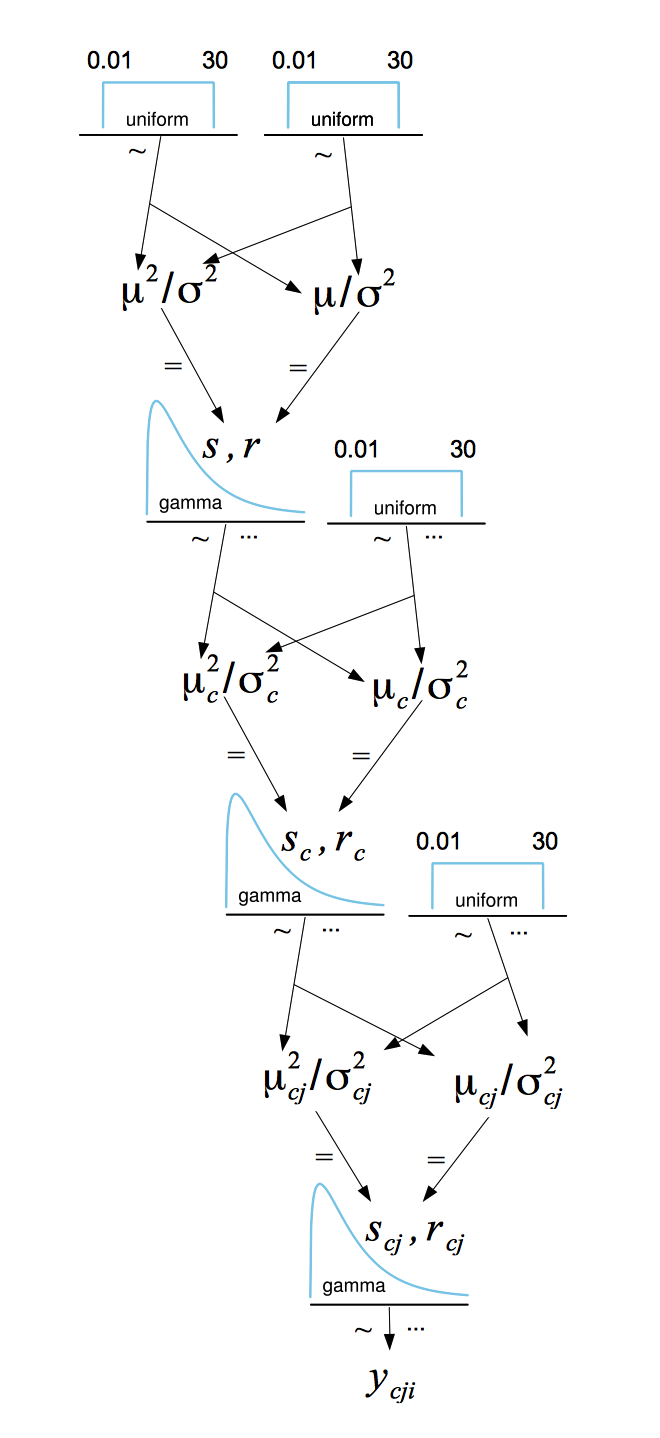

Supplement: S1 Fig — The graphical model, inspired by[25], describes the dependencies in the hierarchical Bayesian model used to estimate response times. The response time y cji from coherence level c, participant j and trial i depend on the shape s cj and rate r cj parameters of a gamma distribution, which are transformed from mean and standard deviation parameters. Parameters for each subject and coherence depend on coherence parameters. Coherence parameters depend on group parameters, which depend on non-informative priors. The "~" symbol describes that values are drawn from the above distributions while the " = " symbol means that values have a deterministic dependency. The ellipsis "…" symbol describes a repeated dependency, i.e. parameters are estimated for multiple coherence levels. (TIFF) [file pone.0140361.s001.tiff]

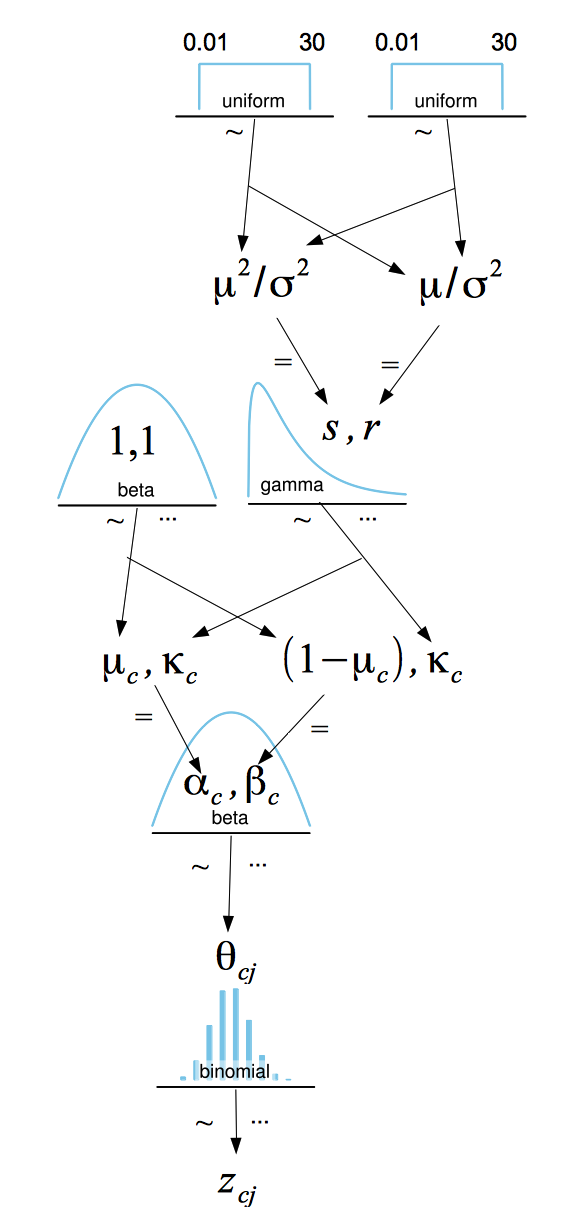

Supplement: S2 Fig — The graphical model, inspired by[25], describes the dependencies in the hierarchical Bayesian model used to estimate correct responses. Number of correct responses z cj of N cj total responses from coherence level c and participant j depend on the value of the θcj parameter in a binomial distribution. Parameters for each subject and coherence depend on condition parameters. Coherence parameters depend on group parameters, which depend on non-informative priors. The "~" symbol describes that values are drawn from a distribution while the " = " symbol means that values have a deterministic dependency. The ellipsis "…" symbol describes a repeated dependency, i.e. parameters are estimated for multiple coherence levels. (TIFF) [file pone.0140361.s002.tiff]

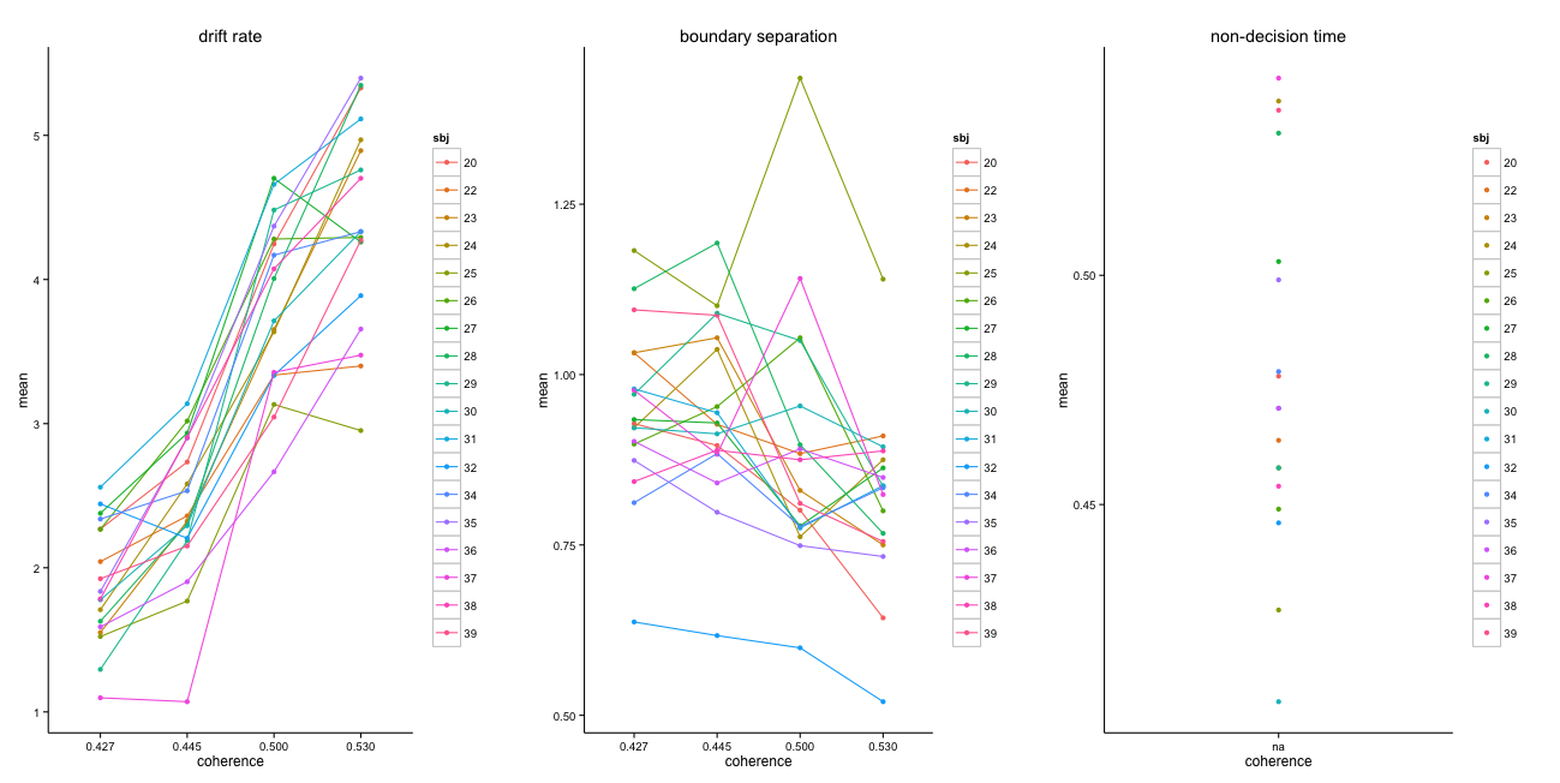

Supplement: S3 Fig — Individual parameter estimates for drift rate, boundary separation and non-decision time across coherence levels (except non-decision time). (TIFF) [file pone.0140361.s003.tiff]

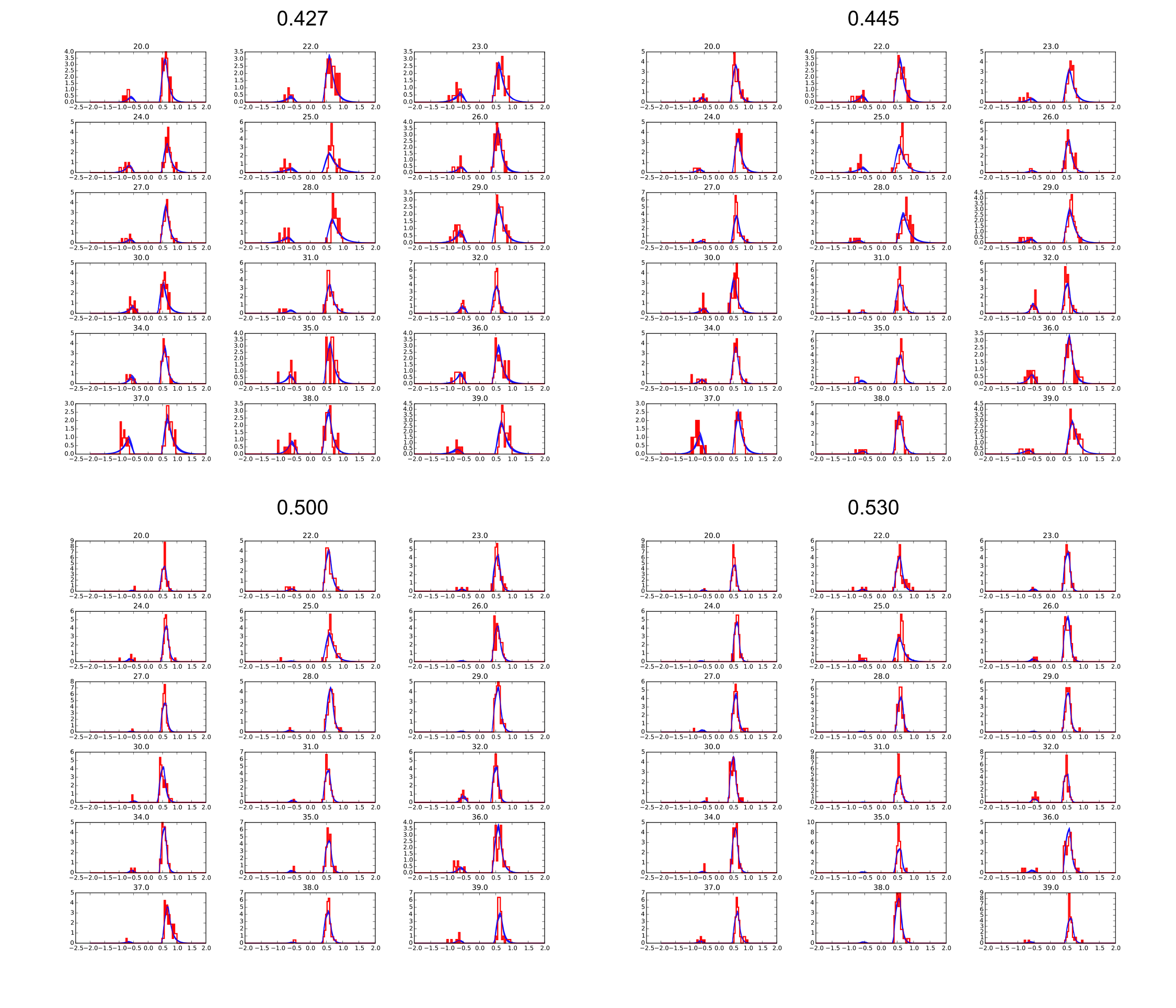

Supplement: S4 Fig — Observed RT distributions (red) for error (coded with negative reaction times) and correct (coded with positive reaction times) responses and predicted response distributions (blue) for each subject based on estimated parameter values across difficulty level. (TIFF) [file pone.0140361.s004.tiff]

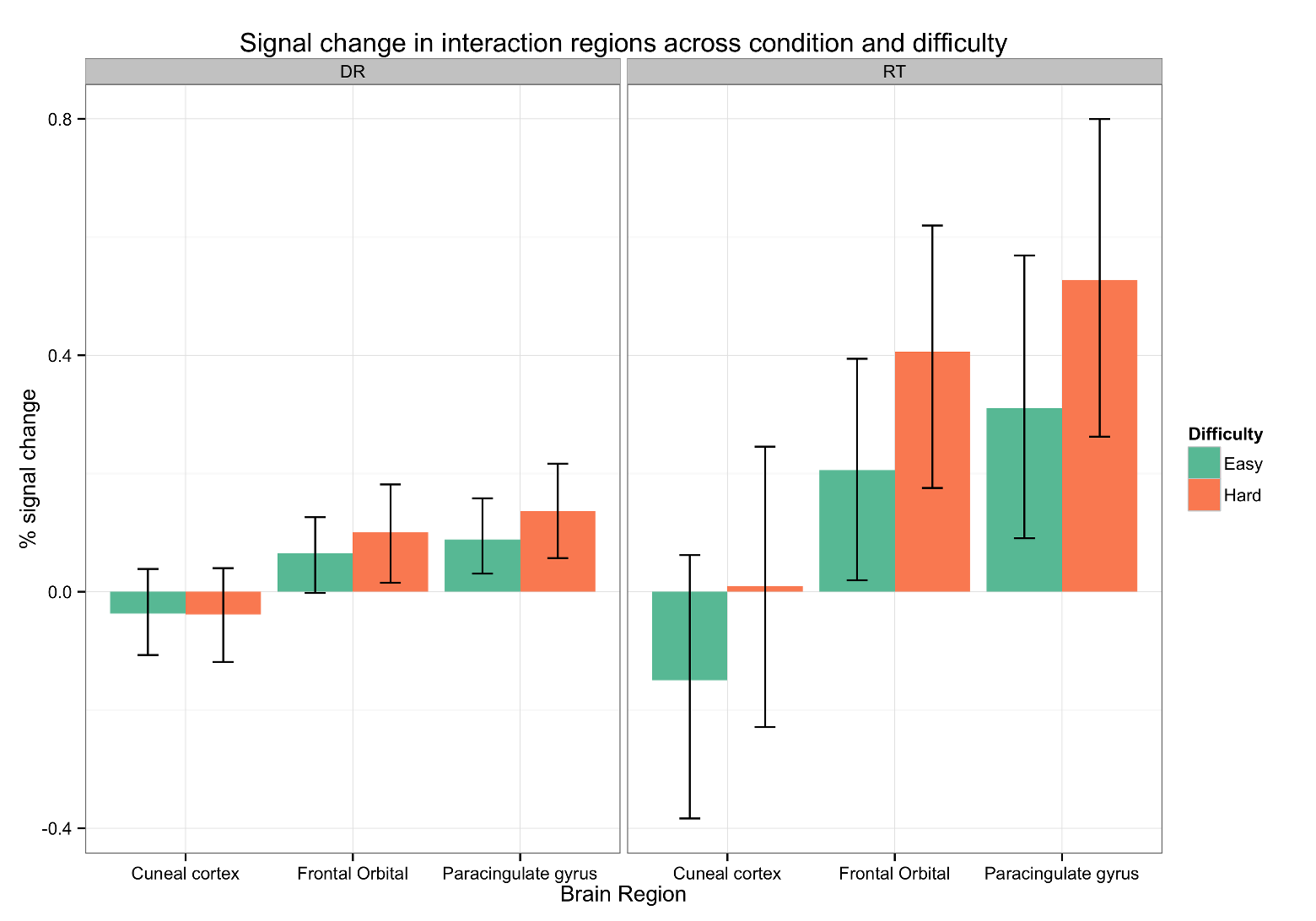

Supplement: S5 Fig — Percent signal change compared to baseline in delayed response (DR) and reaction time (RT) conditions across easy (green) and hard (orange) difficulty levels in clusters identified in interaction analysis (Z>2.3, corrected to p<0.05 at whole-brain level). (TIFF) [file pone.0140361.s005.tiff]

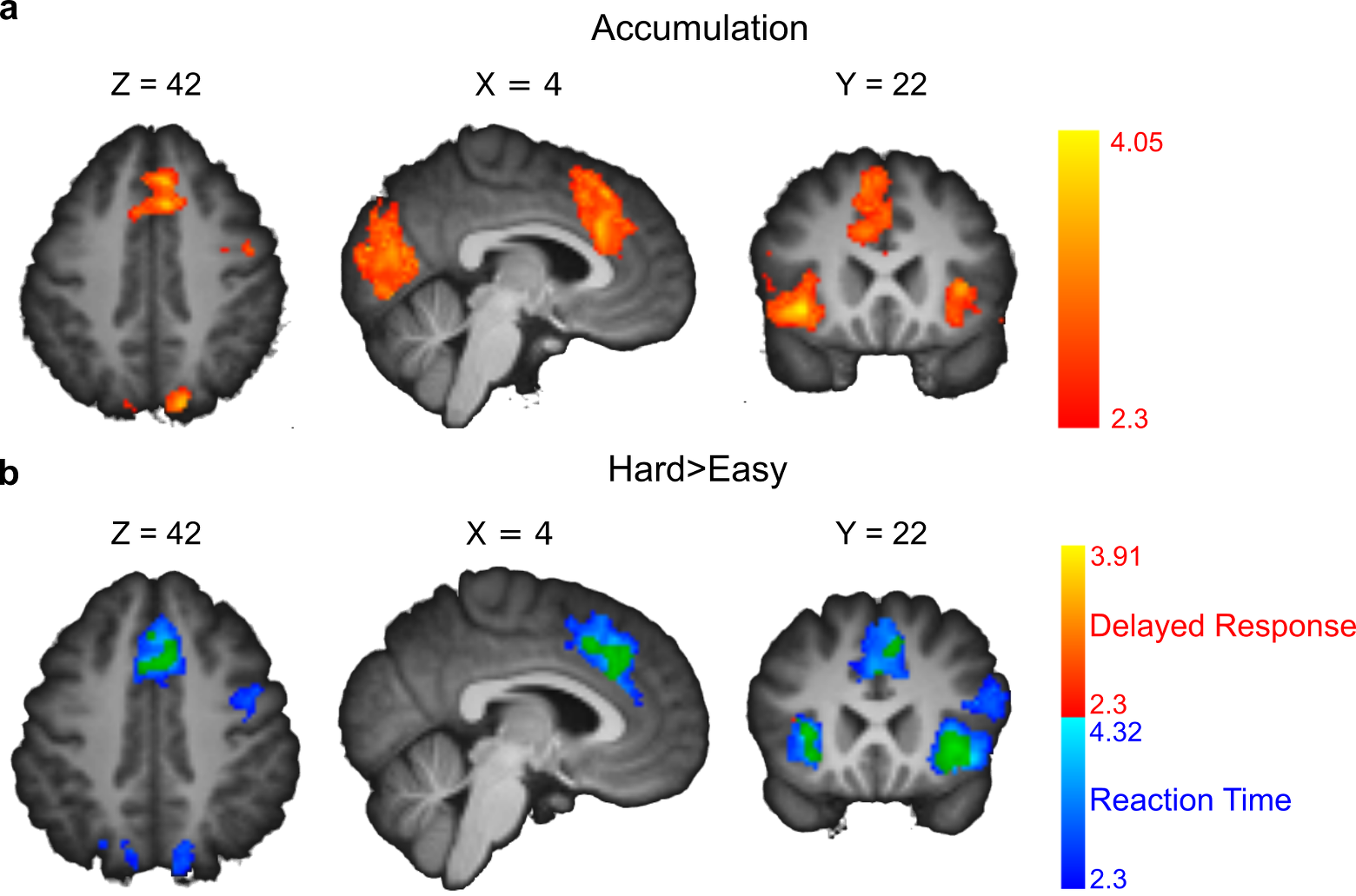

Supplement: S6 Fig — (A) Clusters identified to be negatively correlated with individual estimates of drift rate (Z>2.3, corrected to p<0.05 at whole-brain level) compared with (B) cluster activations for hard>easy contrasts for reaction time (RT) (blue) and delayed response (DR) (red) with overlapping activations in green (Z>2.3, cluster corrected to p<0.05 with 3dClustSim). (TIFF) [file pone.0140361.s006.tiff]

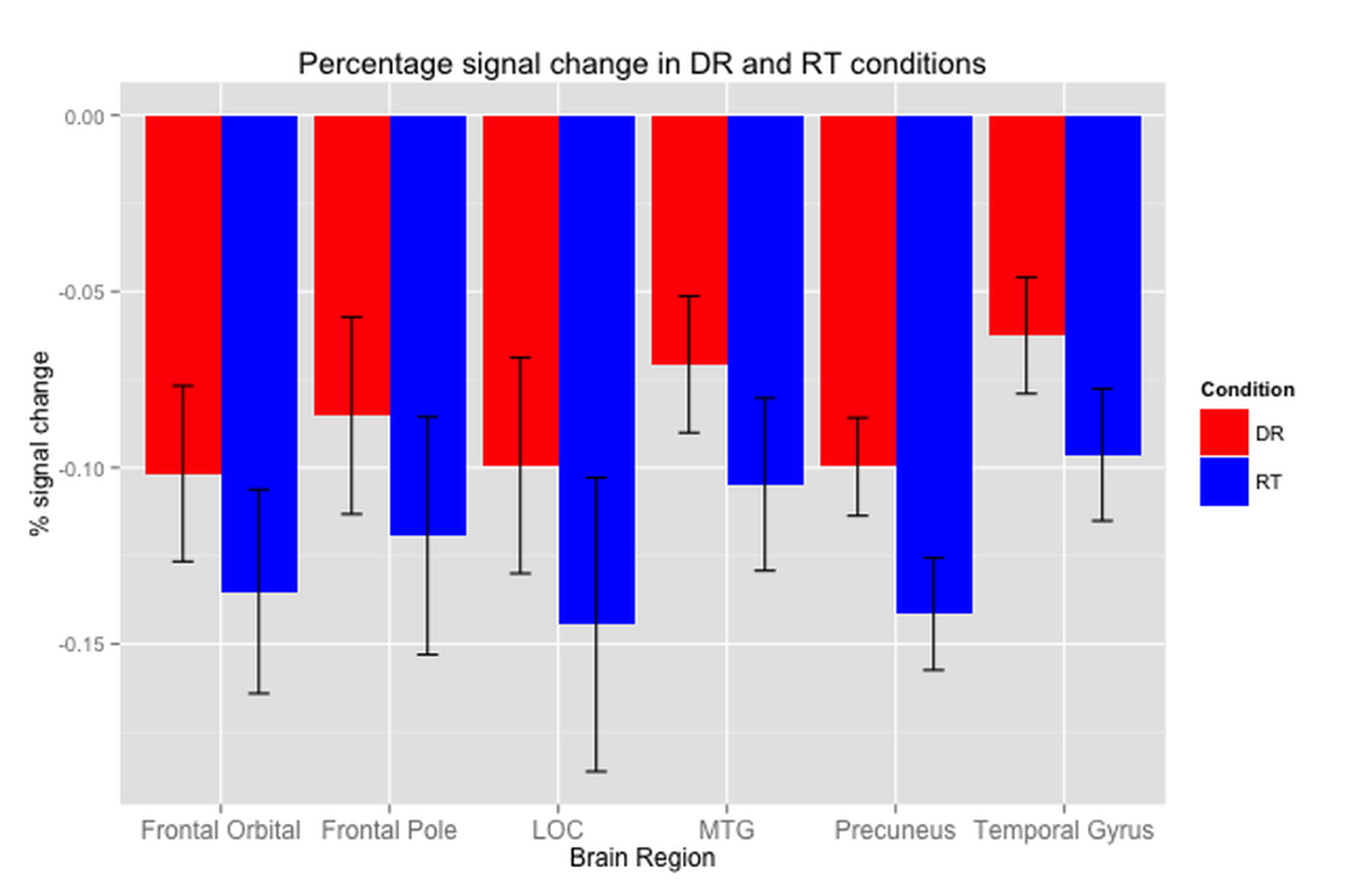

Supplement: S7 Fig — Percent signal change compared to baseline in reaction time (RT) (blue) and delayed response (DR) (red) conditions in clusters activated more in the delayed response than reaction condition. (TIFF) [file pone.0140361.s007.tiff]
