# Supplementary material for: Evidence Accumulation and Choice Maintenance Are Dissociated in Human Perceptual Decision Making
Source: PLoS One. 2015 Oct 28;10(10):e0140361. doi: 10.1371/journal.pone.0140361 (PMC4624809; doi:10.1371/journal.pone.0140361)
Supplement: S1 Table — SD = Standard Deviation. q = quantile. (DOCX) [file pone.0140361.s008.docx]

|  | **parameter** | **subject** | **coherence** | **mean** | **2.5q** | **97.5q** |
| --- | --- | --- | --- | --- | --- | --- |
| **group parameters** |  |  |  |  |  |  |
|  | boundary separation |  | 427 | 0.955 | 0.870 | 1.042 |
|  |  |  | 445 | 0.951 | 0.860 | 1.038 |
|  |  |  | 500 | 0.883 | 0.796 | 0.978 |
|  |  |  | 530 | 0.824 | 0.735 | 0.918 |
|  | boundary separation (SD) |  |  | 0.167 | 0.129 | 0.213 |
|  | drift rate |  | 427 | 1.892 | 1.507 | 2.290 |
|  |  |  | 445 | 2.404 | 2.011 | 2.808 |
|  |  |  | 500 | 3.818 | 3.377 | 4.268 |
|  |  |  | 530 | 4.407 | 3.946 | 4.901 |
|  | inter-trial variability in drift rate |  |  | 0.372 | 0.017 | 0.809 |
|  | drift rate (SD) |  |  | 0.711 | 0.527 | 0.931 |
|  | non-decision time |  |  | 0.478 | 0.458 | 0.500 |
|  | inter-trial variability in non-decision time |  |  | 0.202 | 0.190 | 0.212 |
|  | non-decision time (SD) |  |  | 0.043 | 0.030 | 0.062 |
| **individual parameters** |  |  |  |  |  |  |
|  |  | 20 |  |  |  |  |
|  | boundary separation |  | 427 | 0.928 | 0.788 | 1.084 |
|  |  |  | 445 | 0.896 | 0.771 | 1.045 |
|  |  |  | 500 | 0.801 | 0.653 | 0.982 |
|  |  |  | 530 | 0.643 | 0.492 | 0.822 |
|  | drift rate |  | 427 | 2.269 | 1.556 | 2.998 |
|  |  |  | 445 | 2.734 | 2.027 | 3.489 |
|  |  |  | 500 | 4.246 | 3.330 | 5.232 |
|  |  |  | 530 | 5.329 | 4.231 | 6.546 |
|  | non-decision time |  |  | 0.478 | 0.460 | 0.493 |
|  |  | 22 |  |  |  |  |
|  | boundary separation |  | 427 | 1.032 | 0.899 | 1.195 |
|  |  |  | 445 | 0.927 | 0.800 | 1.069 |
|  |  |  | 500 | 0.884 | 0.752 | 1.042 |
|  |  |  | 530 | 0.910 | 0.770 | 1.073 |
|  | drift rate |  | 427 | 2.043 | 1.400 | 2.678 |
|  |  |  | 445 | 2.360 | 1.676 | 3.079 |
|  |  |  | 500 | 3.337 | 2.572 | 4.096 |
|  |  |  | 530 | 3.400 | 2.631 | 4.191 |
|  | non-decision time |  |  | 0.464 | 0.448 | 0.479 |
|  |  | 23 |  |  |  |  |
|  | boundary separation |  | 427 | 1.032 | 0.907 | 1.173 |
|  |  |  | 445 | 1.054 | 0.906 | 1.225 |
|  |  |  | 500 | 0.830 | 0.691 | 0.981 |
|  |  |  | 530 | 0.750 | 0.594 | 0.928 |
|  | drift rate |  | 427 | 1.551 | 0.983 | 2.128 |
|  |  |  | 445 | 2.323 | 1.697 | 2.960 |
|  |  |  | 500 | 3.653 | 2.802 | 4.549 |
|  |  |  | 530 | 4.894 | 3.889 | 5.999 |
|  | non-decision time |  |  | 0.458 | 0.445 | 0.471 |
|  |  | 24 |  |  |  |  |
|  | boundary separation |  | 427 | 0.922 | 0.806 | 1.063 |
|  |  |  | 445 | 1.037 | 0.893 | 1.206 |
|  |  |  | 500 | 0.762 | 0.640 | 0.905 |
|  |  |  | 530 | 0.875 | 0.686 | 1.100 |
|  | drift rate |  | 427 | 1.709 | 1.037 | 2.364 |
|  |  |  | 445 | 2.582 | 1.911 | 3.248 |
|  |  |  | 500 | 3.637 | 2.782 | 4.553 |
|  |  |  | 530 | 4.969 | 3.952 | 6.059 |
|  | non-decision time |  |  | 0.538 | 0.524 | 0.550 |
|  |  | 25 |  |  |  |  |
|  | boundary separation |  | 427 | 1.182 | 1.023 | 1.364 |
|  |  |  | 445 | 1.101 | 0.971 | 1.253 |
|  |  |  | 500 | 1.435 | 1.192 | 1.744 |
|  |  |  | 530 | 1.140 | 0.962 | 1.348 |
|  | drift rate |  | 427 | 1.523 | 0.930 | 2.102 |
|  |  |  | 445 | 1.769 | 1.216 | 2.324 |
|  |  |  | 500 | 3.133 | 2.429 | 3.890 |
|  |  |  | 530 | 2.952 | 2.249 | 3.698 |
|  | non-decision time |  |  | 0.427 | 0.418 | 0.435 |
|  |  | 26 |  |  |  |  |
|  | boundary separation |  | 427 | 0.898 | 0.771 | 1.042 |
|  |  |  | 445 | 0.953 | 0.809 | 1.120 |
|  |  |  | 500 | 1.054 | 0.864 | 1.295 |
|  |  |  | 530 | 0.800 | 0.656 | 0.970 |
|  | drift rate |  | 427 | 2.267 | 1.609 | 2.965 |
|  |  |  | 445 | 3.018 | 2.266 | 3.815 |
|  |  |  | 500 | 4.281 | 3.379 | 5.251 |
|  |  |  | 530 | 4.292 | 3.388 | 5.221 |
|  | non-decision time |  |  | 0.449 | 0.431 | 0.462 |
|  |  | 27 |  |  |  |  |
|  | boundary separation |  | 427 | 0.934 | 0.805 | 1.081 |
|  |  |  | 445 | 0.929 | 0.782 | 1.090 |
|  |  |  | 500 | 0.778 | 0.614 | 0.970 |
|  |  |  | 530 | 0.863 | 0.711 | 1.046 |
|  | drift rate |  | 427 | 2.378 | 1.712 | 3.057 |
|  |  |  | 445 | 2.934 | 2.188 | 3.695 |
|  |  |  | 500 | 4.702 | 3.704 | 5.836 |
|  |  |  | 530 | 4.260 | 3.367 | 5.182 |
|  | non-decision time |  |  | 0.503 | 0.486 | 0.518 |
|  |  | 28 |  |  |  |  |
|  | boundary separation |  | 427 | 1.126 | 0.972 | 1.291 |
|  |  |  | 445 | 1.193 | 1.025 | 1.391 |
|  |  |  | 500 | 0.897 | 0.742 | 1.085 |
|  |  |  | 530 | 0.767 | 0.592 | 0.979 |
|  | drift rate |  | 427 | 1.629 | 1.063 | 2.205 |
|  |  |  | 445 | 2.304 | 1.705 | 2.903 |
|  |  |  | 500 | 4.007 | 3.105 | 4.956 |
|  |  |  | 530 | 5.347 | 4.227 | 6.619 |
|  | non-decision time |  |  | 0.531 | 0.515 | 0.544 |
|  |  | 29 |  |  |  |  |
|  | boundary separation |  | 427 | 0.971 | 0.856 | 1.095 |
|  |  |  | 445 | 1.090 | 0.946 | 1.253 |
|  |  |  | 500 | 1.050 | 0.855 | 1.279 |
|  |  |  | 530 | 0.836 | 0.677 | 1.039 |
|  | drift rate |  | 427 | 1.295 | 0.744 | 1.865 |
|  |  |  | 445 | 2.188 | 1.535 | 2.817 |
|  |  |  | 500 | 4.482 | 3.591 | 5.500 |
|  |  |  | 530 | 4.760 | 3.761 | 5.811 |
|  | non-decision time |  |  | 0.458 | 0.447 | 0.467 |
|  |  | 30 |  |  |  |  |
|  | boundary separation |  | 427 | 0.922 | 0.810 | 1.041 |
|  |  |  | 445 | 0.913 | 0.786 | 1.054 |
|  |  |  | 500 | 0.954 | 0.801 | 1.127 |
|  |  |  | 530 | 0.894 | 0.742 | 1.073 |
|  | drift rate |  | 427 | 1.778 | 1.157 | 2.393 |
|  |  |  | 445 | 2.292 | 1.595 | 2.984 |
|  |  |  | 500 | 3.713 | 2.886 | 4.577 |
|  |  |  | 530 | 4.333 | 3.438 | 5.286 |
|  | non-decision time |  |  | 0.407 | 0.396 | 0.416 |
|  |  | 31 |  |  |  |  |
|  | boundary separation |  | 427 | 0.979 | 0.836 | 1.148 |
|  |  |  | 445 | 0.944 | 0.798 | 1.113 |
|  |  |  | 500 | 0.775 | 0.619 | 0.955 |
|  |  |  | 530 | 0.837 | 0.655 | 1.059 |
|  | drift rate |  | 427 | 2.559 | 1.822 | 3.307 |
|  |  |  | 445 | 3.138 | 2.387 | 3.893 |
|  |  |  | 500 | 4.660 | 3.717 | 5.731 |
|  |  |  | 530 | 5.114 | 4.085 | 6.317 |
|  | non-decision time |  |  | 0.471 | 0.457 | 0.485 |
|  |  | 32 |  |  |  |  |
|  | boundary separation |  | 427 | 0.637 | 0.522 | 0.761 |
|  |  |  | 445 | 0.617 | 0.509 | 0.734 |
|  |  |  | 500 | 0.599 | 0.489 | 0.718 |
|  |  |  | 530 | 0.520 | 0.415 | 0.635 |
|  | drift rate |  | 427 | 2.443 | 1.595 | 3.352 |
|  |  |  | 445 | 2.205 | 1.339 | 3.050 |
|  |  |  | 500 | 3.335 | 2.387 | 4.264 |
|  |  |  | 530 | 3.888 | 2.878 | 4.912 |
|  | non-decision time |  |  | 0.446 | 0.430 | 0.462 |
|  |  | 34 |  |  |  |  |
|  | boundary separation |  | 427 | 0.812 | 0.694 | 0.944 |
|  |  |  | 445 | 0.884 | 0.760 | 1.028 |
|  |  |  | 500 | 0.777 | 0.635 | 0.943 |
|  |  |  | 530 | 0.834 | 0.684 | 1.012 |
|  | drift rate |  | 427 | 2.338 | 1.582 | 3.100 |
|  |  |  | 445 | 2.534 | 1.826 | 3.253 |
|  |  |  | 500 | 4.169 | 3.236 | 5.155 |
|  |  |  | 530 | 4.332 | 3.428 | 5.283 |
|  | non-decision time |  |  | 0.479 | 0.466 | 0.491 |
|  |  | 35 |  |  |  |  |
|  | boundary separation |  | 427 | 0.874 | 0.762 | 0.999 |
|  |  |  | 445 | 0.798 | 0.669 | 0.946 |
|  |  |  | 500 | 0.749 | 0.600 | 0.913 |
|  |  |  | 530 | 0.733 | 0.572 | 0.929 |
|  | drift rate |  | 427 | 1.836 | 1.202 | 2.492 |
|  |  |  | 445 | 2.900 | 2.105 | 3.742 |
|  |  |  | 500 | 4.370 | 3.414 | 5.413 |
|  |  |  | 530 | 5.397 | 4.327 | 6.588 |
|  | non-decision time |  |  | 0.499 | 0.482 | 0.513 |
|  |  | 36 |  |  |  |  |
|  | boundary separation |  | 427 | 0.902 | 0.795 | 1.022 |
|  |  |  | 445 | 0.841 | 0.733 | 0.966 |
|  |  |  | 500 | 0.891 | 0.765 | 1.027 |
|  |  |  | 530 | 0.849 | 0.713 | 1.005 |
|  | drift rate |  | 427 | 1.590 | 0.971 | 2.224 |
|  |  |  | 445 | 1.903 | 1.215 | 2.599 |
|  |  |  | 500 | 2.666 | 1.918 | 3.437 |
|  |  |  | 530 | 3.656 | 2.841 | 4.481 |
|  | non-decision time |  |  | 0.471 | 0.454 | 0.485 |
|  |  | 37 |  |  |  |  |
|  | boundary separation |  | 427 | 0.977 | 0.868 | 1.102 |
|  |  |  | 445 | 0.883 | 0.775 | 1.006 |
|  |  |  | 500 | 1.141 | 0.960 | 1.363 |
|  |  |  | 530 | 0.824 | 0.696 | 0.968 |
|  | drift rate |  | 427 | 1.590 | 0.971 | 2.224 |
|  |  |  | 445 | 1.071 | 0.439 | 1.709 |
|  |  |  | 500 | 3.356 | 2.609 | 4.131 |
|  |  |  | 530 | 3.475 | 2.668 | 4.295 |
|  | non-decision time |  |  | 0.543 | 0.532 | 0.552 |
|  |  | 38 |  |  |  |  |
|  | boundary separation |  | 427 | 0.843 | 0.737 | 0.963 |
|  |  |  | 445 | 0.889 | 0.756 | 1.047 |
|  |  |  | 500 | 0.875 | 0.715 | 1.065 |
|  |  |  | 530 | 0.888 | 0.716 | 1.088 |
|  | drift rate |  | 427 | 1.784 | 1.122 | 2.443 |
|  |  |  | 445 | 2.904 | 2.160 | 3.681 |
|  |  |  | 500 | 4.074 | 3.219 | 5.014 |
|  |  |  | 530 | 4.702 | 3.767 | 5.706 |
|  | non-decision time |  |  | 0.454 | 0.438 | 0.468 |
|  |  | 39 |  |  |  |  |
|  | boundary separation |  | 427 | 1.095 | 0.939 | 1.283 |
|  |  |  | 445 | 1.087 | 0.945 | 1.253 |
|  |  |  | 500 | 0.811 | 0.687 | 0.957 |
|  |  |  | 530 | 0.755 | 0.609 | 0.921 |
|  | drift rate |  | 427 | 1.924 | 1.253 | 2.610 |
|  |  |  | 445 | 2.151 | 1.548 | 2.741 |
|  |  |  | 500 | 3.046 | 2.240 | 3.865 |
|  |  |  | 530 | 4.272 | 3.354 | 5.283 |
|  | non-decision time |  |  | 0.536 | 0.520 | 0.549 |
